# A shortened verbal autopsy instrument for use in routine mortality surveillance systems

**DOI:** 10.1186/s12916-015-0528-8

**Published:** 2015-12-16

**Authors:** Peter Serina, Ian Riley, Andrea Stewart, Abraham D. Flaxman, Rafael Lozano, Meghan D Mooney, Richard Luning, Bernardo Hernandez, Robert Black, Ramesh Ahuja, Nurul Alam, Sayed Saidul Alam, Said Mohammed Ali, Charles Atkinson, Abdulla H. Baqui, Hafizur R. Chowdhury, Lalit Dandona, Rakhi Dandona, Emily Dantzer, Gary L Darmstadt, Vinita Das, Usha Dhingra, Arup Dutta, Wafaie Fawzi, Michael Freeman, Saman Gamage, Sara Gomez, Dilip Hensman, Spencer L. James, Rohina Joshi, Henry D. Kalter, Aarti Kumar, Vishwajeet Kumar, Marilla Lucero, Saurabh Mehta, Bruce Neal, Summer Lockett Ohno, David Phillips, Kelsey Pierce, Rajendra Prasad, Devarsetty Praveen, Zul Premji, Dolores Ramirez-Villalobos, Rasika Rampatige, Hazel Remolador, Minerva Romero, Mwanaidi Said, Diozele Sanvictores, Sunil Sazawal, Peter K. Streatfield, Veronica Tallo, Alireza Vadhatpour, Nandalal Wijesekara, Christopher J. L. Murray, Alan D. Lopez

**Affiliations:** Institute for Health Metrics and Evaluation, University of Washington, 2301 Fifth Ave., Suite 600, Seattle, WA 98121 USA; University of Queensland, School of Public Health, Level 2 Public Health Building School of Public Health, Herston Road, Herston, QLD 4006 Australia; National Institute of Public Health, Av. Universidad 655, Buena Vista, 62100 Cuernavaca, Morelos Mexico; Institute for International Programs, Johns Hopkins University, Bloomberg School of Public Health, 615 N Wolfe St., Baltimore, MD 21205 USA; Community Empowerment Lab, Shivgarh, India; The INCLEN Trust International, New Delhi, India; International Center for Diarrhoeal Disease Research, Dhaka, Bangladesh; Public Health Laboratory-IdC, P.O.BOX 122, Wawi, Chake Chake, Pemba, Zanzibar Tanzania; Public Health Foundation of India, Plot 47, Sector 44, Gurgaon, 122002 National Capital Region India; Malaria Consortium Cambodia, 113 Mao Tse Toung, Phnom Penh, Cambodia; Department of Pediatrics, Stanford University School of Medicine, Stanford, CA 94304 USA; CSM Medical University, Shah Mina Road, Chowk Lucknow, Uttar Pradesh 226003 India; Harvard School of Public Health, 677 Huntington Avenue, Boston, MA 02115-6018 USA; WHO Collaborating Centre for Public Health Workforce Development, National Institute of Health Sciences, Kalutara, Sri Lanka; Iipas, Chapel Hill, NC USA; The George Institute for Global Health, Sydney, Australia; Research Institute for Tropical Medicine, Corporate Ave., Muntinlupa City, 1781 Philippines; Cornell University, Division of Nutritional Sciences, 314 Savage Hall, Ithaca, NY 14853 USA; The George Institute for Global Health, University of Sydney and Royal Prince Albert Hospital, Sydney, Australia; Imperial college, London, London, UK; The George Institute for Global Health, Hyderabad, India; Muhimbili University of Health and Allied Sciences, United Nations Rd., Dar es Salaam, Tanzania; University of Melbourne, School of Population and Global Health, Building 379, 207 Bouverie St., Parkville, 3010 VIC Australia

**Keywords:** Verbal autopsy questionnaire, Mortality surveillance, Causes of death

## Abstract

**Background:**

Verbal autopsy (VA) is recognized as the only feasible alternative to comprehensive medical certification of deaths in settings with no or unreliable vital registration systems. However, a barrier to its use by national registration systems has been the amount of time and cost needed for data collection. Therefore, a short VA instrument (VAI) is needed. In this paper we describe a shortened version of the VAI developed for the Population Health Metrics Research Consortium (PHMRC) Gold Standard Verbal Autopsy Validation Study using a systematic approach.

**Methods:**

We used data from the PHMRC validation study. Using the Tariff 2.0 method, we first established a rank order of individual questions in the PHMRC VAI according to their importance in predicting causes of death. Second, we reduced the size of the instrument by dropping questions in reverse order of their importance. We assessed the predictive performance of the instrument as questions were removed at the individual level by calculating chance-corrected concordance and at the population level with cause-specific mortality fraction (CSMF) accuracy. Finally, the optimum size of the shortened instrument was determined using a first derivative analysis of the decline in performance as the size of the VA instrument decreased for adults, children, and neonates.

**Results:**

The full PHMRC VAI had 183, 127, and 149 questions for adult, child, and neonatal deaths, respectively. The shortened instrument developed had 109, 69, and 67 questions, respectively, representing a decrease in the total number of questions of 40-55 %. The shortened instrument, with text, showed non-significant declines in CSMF accuracy from the full instrument with text of 0.4 %, 0.0 %, and 0.6 % for the adult, child, and neonatal modules, respectively.

**Conclusions:**

We developed a shortened VAI using a systematic approach, and assessed its performance when administered using hand-held electronic tablets and analyzed using Tariff 2.0. The length of a VA questionnaire was shortened by almost 50 % without a significant drop in performance. The shortened VAI developed reduces the burden of time and resources required for data collection and analysis of cause of death data in civil registration systems.

**Electronic supplementary material:**

The online version of this article (doi:10.1186/s12916-015-0528-8) contains supplementary material, which is available to authorized users.

## Background

Cause of death (COD) information is essential to guide and inform health policy and priority debates [1]. Ideally, COD data would be based on accurate medical certification and registration of all deaths [2]. However, vital registration systems still function poorly in many countries, particularly in resource-poor settings where mortality rates are higher and accurate cause of death information is most crucial [3]. Verbal autopsy (VA) is now becoming recognized as the only feasible alternative to comprehensive medical certification of deaths in such settings. The World Health Organization has now called for wider use of VA to improve understanding of the causes of mortality and the nature of mortality change in national populations [4].

Although VAs have been incorporated into official data collection systems already in place in countries such as India [5], Brazil [6], Bangladesh [7], and Sri Lanka [8], as well as through the collection of VA samples during national censuses as in Mozambique [9], doubts have remained about the ability of VAs to provide accurate and timely information about the COD in populations. This can be attributed, in large part, to the initial reliance on physician certification of verbal autopsies (PCVA) in demographic and health surveillance research sites. PCVA is time-consuming and expensive, and it is difficult to maintain the quality of cause assignment on a large scale over long periods of time.

These problems, however, can be resolved by introducing automated VA diagnostic methods, which have been shown to out-perform PCVA in terms of their accuracy. They now offer the potential for inexpensive, rapid, and reliable COD assignments for deaths occurring outside of hospitals [10–13].

Current practice in the application of VA is to collect interview information using paper-based verbal autopsy instruments (VAIs), which have been largely derived from VA methods developed for research sites in the 1980s and 1990s [14, 15]. A barrier to their widespread adoption by national registration systems has been the amount of time and, hence, cost needed to conduct interviews and to maintain their quality. For widespread application, a short instrument is needed, but one that still enables automated diagnostic systems to make accurate predictions of causes of death. At the same time, electronic systems for data collection need to replace paper-based systems.

We address these needs in this paper and describe a shortened version of the VAI developed for the Population Health Metrics Research Consortium Gold Standard Verbal Autopsy Validation Study (PHMRC study) [16]. Using a formal empirically-based method to shorten the VAI, we identify the key survey questions and the optimal length of a shortened VAI.

## Methods

Our general approach was first to establish a rank order of individual question items in the PHMRC VAI in terms of their importance in predicting COD. We did this using the Tariff 2.0 Method [17] to predict the COD for each VA in the PHMRC Gold Standard database and by comparing the predicted COD with the gold standard cause. Second, we reduced the size of the instrument by dropping items in reverse order of their importance. We assessed the predictive performance of the instrument at each stage of item reduction by calculating chance-corrected concordance (CCC) at the level of the individual and cause specific mortality fraction (CSMF) accuracy at the level of the population (see below). Finally, the optimum size of the shortened instrument was determined using a first derivative analysis of the decline in performance as the size of the VAI progressively decreased. We followed the same approach for adults, children, and neonates.

### PHMRC gold standard validation study database

The general methodology of the PHMRC study has been described in detail elsewhere [16]. In summary, VAs were collected from six sites in four countries: Andhra Pradesh and Uttar Pradesh in India, Bohol in the Philippines, Mexico City in Mexico, and Dar es Salaam and Pemba Island in Tanzania. Methods were approved by the Internal Review Boards of the University of Washington, Seattle, WA, USA; School of Public Health, University of Queensland, Australia; George Institute for Global Health, Hyderabad, India;  National Institute  of Public Health, Mexico;  Research Institute for Tropical Medicine, Alabang, Metro Manila, Philippines; Muhimbili University, Tanzania; Public Health Laboratory Ivo de Carneri, Tanzania; and  CSM Medical University, India. All data were collected with prior informed consent. Gold standard clinical diagnostic criteria for hospital deaths were specified for an initial list of 53 adult, 27 child, and 13 neonatal causes including stillbirths, chosen on the basis of epidemiological criteria and the likely ability of VA to identify the cause (Additional file 1). This was known as the target cause list. Deaths with hospital records fulfilling the gold standard criteria were identified in each of the sites. The PHMRC VAI was used to interview families about the events leading to each of these deaths [16]. Interviewers were blinded to the COD assigned in the hospital. The PHMRC database contains 12,501 verbal autopsies with gold standard diagnoses (7,846 adults, 2,064 children, 1,586 neonates, and 1,005 stillbirths).

The PHMRC VAI includes both closed-ended questions and an open-ended narrative. Questions covered: 1) symptoms of the terminal illness, 2) diagnoses of chronic illnesses obtained from health service providers, 3) risk behaviors (tobacco and alcohol), 4) details of any interactions with health services, and 5) details about the background of the decedent and about the interview itself. Not all of these questions contributed to prediction of the COD. Questions that were converted to binary variables – the necessary basis for Tariff analysis and the prediction of COD – we refer to as question items. Text items were derived from open-ended narrative using a text mining procedure (Text Mining package in R (version 2.14.0) [18]), which identifies keywords and groups words with the same or similar meanings. Performance in this paper is reported as being 1) with text, 2) without text, and 3) with a checklist. The checklist uses only a selected subset of text items as described later.

### Tariff 2.0

The Tariff Method is based on a simple additive algorithm that creates a score, or tariff, for each questionnaire item and uses these scores to assign COD [10, 17]. Ideally, an item would have a high tariff for just one COD and a low tariff for all others; the model would then differentiate readily between causes [10]. For example, the item “Decedent suffered drowning” has a strong association with a few causes of death (accidental drowning, homicide, and suicide) and carries high tariffs for those causes. On the other hand, the item “Decedent had a fever” is associated with many different causes of death and carries low tariffs for the causes it is associated with. Tariffs for drowning have high standard deviations, while tariffs for fever have low standard deviations. Items with high standard deviations were considered more important for diagnosis than were tariffs with low standard deviations. To determine their order of importance, items were ranked by standard deviation. This was done separately for each module (adult, child, and neonate).

### Measurement of performance

#### Simulated populations

The performance of a VA method in assigning a COD is a function of the true cause of death composition in the study population [19]. Therefore, for the development of a VA diagnostic method or a new VAI it is important to validate the method or instrument in as many populations with different cause compositions as possible. This is made practicable by means of computer simulation: 500 populations with random cause compositions were created based on the PHMRC dataset for the development and validation of the original suite of VA methods [16]. In the present study, every test of performance of different length instruments was done using the same 500 randomly generated populations. The 500 train-test data analysis datasets were generated by holding 75 % of the dataset as “training” data and 25 % as “test” data. Each test dataset was resampled using a Dirichlet distribution to obtain a random CSMF composition for each simulated population. Training data were used to generate the model. Analysis of test data was blinded to the gold standard COD. The accuracy of COD predictions was assessed using the performance metrics. This process is described more fully in Additional file 2.

#### Performance metrics

For policy, research, and surveillance it is important to be able to quantify the actual performance of a VA method in predicting the COD, correcting for chance at both individual and population levels. We assessed performance of the progressively shortened VAI using Cohen’s Kappa, CCC, sensitivity, specificity and CSMF accuracy.

CCC measures sensitivity adjusted for chance and was used to assess the extent to which Tariff 2.0 correctly predicted an individual cause of death when applied to the shortened VAI. A perfect prediction has CCC equal to one, while a random allocation would have, on average, CCC equal to zero. CCC is calculated as follows:

$$ CC{C}_j=\frac{\left(\frac{T{P}_j}{T{P}_j+F{N}_j}\right)-\left(\frac{1}{N}\right)}{1-\left(\frac{1}{N}\right)} $$

where TPj is true positives, or the number of decedents with gold standard cause j assigned correctly to cause j, FNj is false negatives, or the number of decedents incorrectly assigned to cause j, and N is the number of causes analyzed. The sum of TPj and FNj is the total number of deaths due to cause j.

Performance was also measured at the population level using mean CSMF accuracy across the 500 cause compositions, calculated as

$$ \mathrm{CSMF}\ \mathrm{accuracy}=1-\frac{{\displaystyle {\sum}_{j=1}^k\left|{\mathrm{CSMF}}_j^{true}-{\mathrm{CSMF}}_j^{pred}\right|}}{2\left(1-\mathrm{Minimum}\left({\mathrm{CSMF}}_j^{true}\right)\right)} $$

where the numerator in the calculation is the sum of the absolute error for all k causes between the true CSMF and estimated CSMF, and the denominator is the maximum possible error across all of the causes. CSMF accuracy will be one when the CSMF for every cause is predicted with no error.

### Developing a shortened verbal autopsy instrument

To begin, we removed questions about the background of decedents from the full PHMRC VAI. We then turned the remaining questions into binary indicators, or items, as described above. Thus, 183 adult, 127 child, and 149 neonatal questions were converted into 170, 80, and 117 question items, respectively. Next, we ranked these items (1–170, 1–80, and 1–117) according to their importance, as defined by the standard deviation of their tariffs. We then systematically reduced the size of the instrument by 10 question items at a time in the order of their importance, as ranked by their tariff standard deviations. With each successive reduction in the number of items, we measured both CCC and CSMF accuracy using the 500 simulated populations as described above. We analyzed the performance of question items with and without text to assess the importance of text as the number of question items decreased. We then used a cubic spline to interpolate between these CCC and CSMF accuracy values to derive a continuous performance curve. Based on this curve, we identified the points (i.e., residual number of items) where each of the metrics (CCC with text, CCC without text, CSMF accuracy with text, CSMF accuracy without text) began to decrease at a significantly negative rate. This was done by taking the first derivative of the continuous performance curves for both CCC and CSMF accuracy. The optimum size of the shortened VAI for each of the three age groups was determined by the number of items that immediately preceded any significant decrease for at least one of these four metrics. These items, which had been ranked in order of importance, formed the basis for the final shortened VAI.

To complete the VAI, we also added questions that would enable the shortened version to function as a stand-alone instrument in a survey. In particular, we inserted questions to preserve the sense and flow of the instrument: for example, an important question was, “Did [name] cough blood?” but this needed to be preceded by the question, “Did [name] have a cough?” We also retained questions relevant to health service utilization and decedent background.

We then piloted the shortened VAI in three sites in the Philippines, Sri Lanka, and Bangladesh to assess its logic and applicability using Android tablets and the open source software, Open Data Kit (ODK) [20].

### Checklist for open narrative

The Tariff Method uses a set of the top-ranked 40 items for each cause prediction based on standard deviation of each item’s tariff [10]. In Tariff 2.0, 43 % of items used in the prediction of all 34 causes in adults were text items derived from open narrative that had been translated into English [17]. We, therefore, concluded that it was critical that we include open narrative in the shortened form of the instrument. We found, however, that we had failed to take into account the difficulties that interviewers would experience in entering open narrative directly onto the tablet. This was a consequence not only of shifting between languages but also between Bengali and Sinhala scripts and the Latin script used for English. During the field trial, some field staff had taken notes on paper, which they transcribed in the office to record the open narrative section. This process took more time and effort than any other component of data management and was a potential source of error. Such difficulties were compounded by the limited character sets for non-Latin scripts on the tablets and the much more extensive training required to enter lengthy text data into a tablet. We, therefore, developed a checklist of keywords to use in the open narrative rather than having interviewers record and transcribe an entire conversation.

This checklist comprised a list of words that were endorsed by the interviewer when mentioned by the respondent in describing the circumstances surrounding the death. These words could be converted directly into English and subjected to text mining.

Using the 500 simulated populations we measured the independent effect on performance of the addition of single text items to the shortened VAI: i.e., on CCC overall, on CCC by cause, and on CSMF accuracy (all question items plus a single text item). This was done separately for the adult, child, and neonate modules. The length of the final checklist for each of the three modules was decided on practical grounds: the checklist needed to fit on a single screen and could not have more words than could easily be remembered by the interviewer during the conversation. It was thus limited to a maximum of 12 text items. The final selection was based both on the items’ contributions to performance and on their significance for the diagnosis of diseases of public health importance.

## Results

The full PHMRC VAI had 183, 127, and 149 questions for adult, child, and neonatal deaths, respectively [21]. The shortened PHMRC VAI developed through this analysis had 109, 69, and 67 questions for adult, child, and neonatal deaths, respectively, representing a decrease in the total number of questions of 40-55 % (Table 1). The paper-based version of the shortened PHMRC VAI is given in Additional file 3. The electronic version, which was created using ODK for installation on Android devices, can be obtained upon request.

**Table 1:** Characteristics of shortened PHMRC verbal autopsy questionnaire (VAI)

	Adult	Child	Neonate
PHMRC VAI - question number	183	127	149
Shortened VAI - question number	109	69	67
% reduction in questionnaire size	40 %	46 %	55 %

**Fig. 1:**
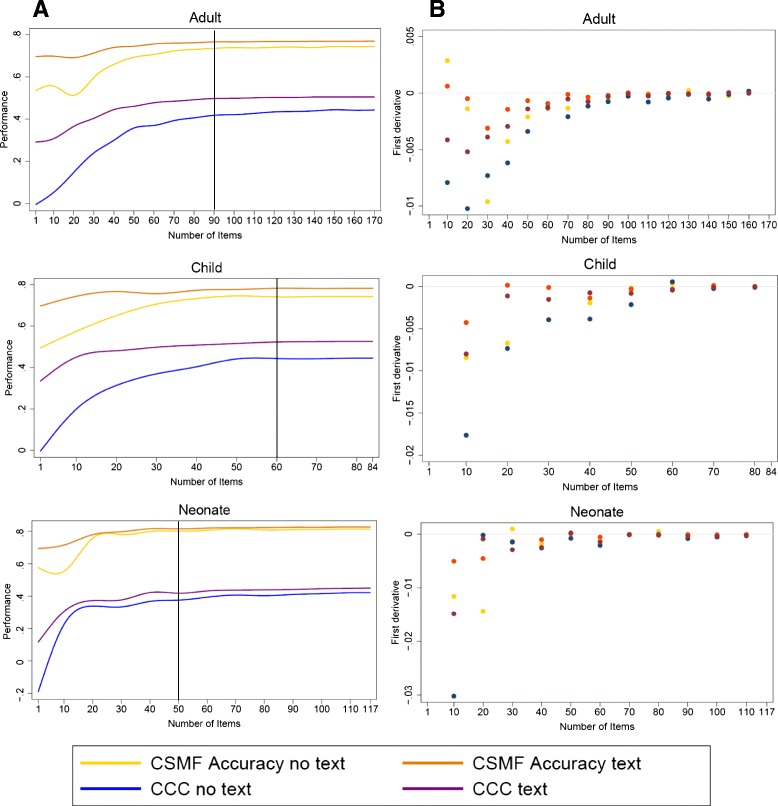
**a**: Decrease in CSMF accuracy and CCC with progressive reduction in the number of question items for each age-specific module, with and without text items. **b**: First derivative of the predictive performance curves for CCC and CSMF accuracy, with and without text items. *CSMF* cause specific mortality fraction, *CCC* chance-corrected concordance

The reduced VAI analyzed with the Tariff 2.0 method ascertains causes of death in 34 mutually exclusive, collectively exhaustive categories for adults, 21 for children, and 6 for neonates, as the original PHMRC VAI. As would be expected, in terms of CCC and CSMF accuracy, the predictive performance of successively shortened versions of the questionnaire declined as question items were systematically removed (Fig. 1a). Performance metrics are shown in Table 2 for questionnaires both with and without text. Sensitivity and specificity for each COD with the long and shortened version of the questionnaire are presented in Additional files 4 and 5. For example, the shortened child module reduced to 69 questions had a CSMF accuracy of 78.3 % when all text items were included and 74.5 % when text items were excluded. CCC for the shortened child module was 52.5 % with text and 44.5 % without. These absolute differences of 3.5 % and 8.0 %, respectively, in diagnostic accuracy are reasonably substantial and continued to increase more or less monotonically as the number of question items was reduced, with the notable exception of CSMF accuracy for adult and neonate deaths without text items in the VAI.

**Table 2:** Chance-corrected concordance and CSMF accuracy for full Population Health Metrics Research Consortium verbal autopsy instrument (PHMRC VAI) as compared to the shortened PHMRC VAI by type of text items included in the analysis

			Chance-corrected concordance		CSMF accuracy	
			Median (%)	95 % CI	Median (%)	95 % CI
Adult	Full PHMRC VAI	No Text	44.3	(44.1, 44.4)	74.5	(74.0, 74.8)
		Text	50.5	(50.3, 50.8)	77	(76.6, 77.5)
	Shortened PHMRC VAI	No Text	43.3	(43.1, 43.5)	74.6	(74.0, 75.2)
		Checklist	46.6	(46.5, 46.8)	76.2	(75.7, 76.7)
		Text	50	(49.8, 50.2)	76.6	(76.2, 77.2)
Child	Full PHMRC VAI	No Text	44.6	(44.2, 45.0)	74.3	(73.6, 75.2)
		Text	52.6	(52.2, 53.1)	78.3	(77.6, 78.7)
	Shortened PHMRC VAI	No Text	44.5	(44.1, 45.0)	74.5	(73.7, 75.2)
		Checklist	51.8	(51.4, 52.1)	78	(77.5, 78.7)
		Text	52.5	(52.2, 52.9)	78.3	(77.6, 78.9)
Neonate	Full PHMRC VAI	No Text	42.2	(41.9, 42.6)	81.4	(80.5, 82.4)
		Text	44.7	(44.3, 45.0)	82.8	(81.9, 83.5)
	Shortened PHMRC VAI	No Text	40.2	(40.0, 40.5)	81.1	(80.1, 81.8)
		Checklist	43.4	(43.0, 43.8)	82.1	(81.4, 83.0)
		Text	43.4	(43.1, 43.6)	82.2	(81.4, 83.1)

*CSMF* cause specific mortality fraction, *CI* confidence interval

Perhaps a better way to summarize the decline in predictive performance with a progressively shorter VAI is to examine the first derivative at different numbers of question items (Fig. 1b). Since the number of question items for the shortened instrument was chosen to maintain performance in terms of CCC and CSMF accuracy, both with and without text items, the first derivative of the curve of performance vs. the number of question items should not drop significantly below zero. Applying this criterion, we identified the optimum number of question items as 90, 60, and 50 for adults, children, and neonates, respectively. This translated into 91, 50, and 48 questions, respectively. It should be noted that significant drop-off in performance of the neonatal module was not seen until there were only about 25 to 30 question items remaining. This reflects the shorter list of target causes for neonates.

As described in the methods section, questions that would enable the shortened version to function as a stand-alone instrument in a survey of importance were added back into the survey. This process increased the number of questions for the short VA to 109, 69, and 67 questions for adults, children, and neonates, respectively.

### Comparative performance

A more detailed assessment of the comparative performance of the shortened vs the longer (original) PHMRC instrument for various text inclusions is given in Table 2. Within modules, CSMF accuracy varied little between different versions of the instrument. The shortened instrument, with text, showed only minor declines in CSMF accuracy from the full instrument with text of 0.4 %, 0.0 %, and 0.6 % for the adult, child, and neonatal modules, respectively. The short instrument with checklist performed slightly worse than the shortened instrument with text, with a decline 0.4 % (adults), 0.3 % (children), and 0.1 % (neonates). The short instrument without text items showed non-significant changes from the full instrument.

Performance at the individual level overall, as assessed by CCC, shows more variation: the average drop in CCC by using the shortened version, with text, was 0.63 %. In the case of short and long versions without text, the average decline in accuracy across the three modules was 1.0 %. This difference was greatly reduced when the checklist was applied.

More generally, the addition of the checklist (Table 3) increased CMSF accuracy and mean CCC by an average of 2.0 % and 4.6 %, respectively. The impact of the checklist on performance was most substantial for the child module (Table 2).

**Table 3:** List of keywords used as a checklist in the open narrative for the adult, child, and neonatal modules

Neonate	Child	Adult
Asphyxia (lack of oxygen)	Abdomen	Chronic kidney disease
Incubator	Cancer	Dialysis
Lung problems	Dehydration	Fever
Pneumonia	Dengue fever	Heart attack (AMI)
Preterm delivery	Diarrhea	Heart problems
Respiratory distress	Fever	Jaundice (yellow skin or eyes)
	Heart problems	Kidney (renal) failure
	Jaundice (yellow skin or eyes)	Liver failure
	Rash	Malaria
		Pneumonia
		Suicide

More specifically, the addition of keywords with the checklist had a significant impact on performance. For example, in adults, the mean CCC declined from 50.5 % in the full instrument with text to 43.3 % in the shortened instrument without text (see also Table 2). Addition of the single text item “malaria” increased overall CCC from 43.3 % to 43.7 %. At the same time, CCC for malaria increased from 34.1 % to 42.3 %. Addition of the text item, “malaria”, also increased CSMF accuracy from 74.6 % in the shortened instrument with no text to 74.7 %.

Addition of the checklist to the shortened instrument without text increases CCC for pneumonia, suicide, and tuberculosis to levels at or above those obtained from the full instrument with text. CCC for cirrhosis and malaria is substantially increased from the short instrument with no text. More detail about the effect of applying various combinations of the shortened questionnaire with text and checklist on CCC for adults, children, and neonates can be found in Additional file 6 for a comprehensive list of causes.

It should be noted that the tariff for a text item was frequently higher than the tariff for the corresponding question item. This reflected lower endorsement rates for text items than for question items. For example, 49.3 % of interviewees overall reported the presence of fever in response to a question but only 13.0 % of all interviewees reported the presence of fever in open-ended narrative. On the other hand, in cases where the gold standard cause of death was malaria, 86.0 % of interviewees reported the presence of fever in response to the question and 51.0 % reported the presence of fever in open-ended narrative. As a consequence, the text item “fever” scored a tariff of 4.0 for malaria but the corresponding question item scored a tariff of only 1.5. A symptom elicited without prompting was more important for diagnosis than a symptom elicited with prompting.

## Discussion

It is difficult to see how substantial improvements in obtaining information about COD patterns in resource-poor populations can be achieved through expanding medical certification. Accurate certification depends on the physician being intimately familiar with the decedent’s clinical history and/or the details of the terminal illness. In resource-poor countries, only a small proportion of decedents have had access to a physician; this situation is unlikely to change in the medium term. If a physician has not been directly involved in the management of a patient, the COD on the person’s medical certificate has little more value than the COD obtained from an unstructured VA (a VA that does not follow a structured and validated format). The routine use of VA with known performance characteristics for all deaths that have not been attended by a physician is the only cost-effective means of obtaining information about cause of death patterns and about how these patterns are changing.

In the context of the PHMRC validation study, in which VAs and diagnosis methods were compared with gold standard hospital deaths, automated computer diagnosis of VAs was shown to be more accurate than PCVA [22]. Reliance on PCVA is inefficient and leads to unnecessary competition for resources with clinical services, often leading to long delays in diagnosing VAs. For example, the VA data collected in India’s Sample Registration System starting in 2002 have still not been reported or released because of delays to physician coding [23]. Outstanding barriers to widespread use are the length of time required to administer the full-length VAI (50–70 minutes) and the resources required for data entry of paper-based records of interview.

The Tariff Method was developed and validated using hospital gold standard deaths [10]. The Method is additive in the sense that it sums tariff scores by cause and by symptom to arrive at the most probable cause for an individual death. The use of hospital gold standard deaths as reference ensured that a full range of symptoms was available for the development of the tariff scores. The process of item-reduction was designed to remove from the questionnaire those symptoms which were not contributing significantly to the summed tariff scores. Tariff 2.0 sets cut-off points to establish that sufficient symptoms are available to assign a COD for an individual death [17]. Such cases are assigned to an indeterminate category under particular circumstances. If the symptom list in the item-reduced instrument were too short this would manifest itself in an increase in the size of the Indeterminate category.

In this paper, we have established the theoretical basis for the validity of a shortened VAI administered by means of hand-held electronic tablets. Although the shortening of an instrument may lead to a decrease in performance and some loss of specificity, at least for rare diseases, we have demonstrated by formal statistical methods applied to validation datasets, where the true COD is known, that it is possible to reduce the length of a VA questionnaire by 40 % or more without a significant drop in performance. The performance characteristics of the shortened VAI are now established. We have also shown that many of the advantages of an open-ended narrative in improving performance can be retained by use of an on-screen checklist. The application of this checklist will require special training of fieldworkers. It is worth highlighting that symptoms mentioned in the open narrative may have a different Tariff score for a given cause of death than their counterparts in the structured questionnaire. This may reflect the fact that more salient information may be easier to be mentioned spontaneously than being recalled after prompted in a questionnaire. The checklist of keywords from the open narrative is, therefore, an important innovation of this shortened questionnaire. It reduces the burden on the interviewer by registering keywords instead of registering the answer verbatim, and also captures these key items that have a substantial contribution to determine the cause of death.

We consider the greatest utility of the shortened VAI will be for the collection of COD data in civil registration systems and for the calculation of CSMFs in populations. To put our results in perspective: a CSMF accuracy of 76.2 % in adults, using the tablet with check list for data entry, compares with a reported CSMF accuracy of 82 % for medical certification of adult deaths in Mexican teaching hospitals [24]; the former equates to an adjusted accuracy of 32.8 % and the latter to an adjusted accuracy of 50 %.

We have set the number of questions items in the shortened VAI at 90 items for adults, 60 for children, and 50 for neonates. We justified the thresholds at the analytical level for question items for each questionnaire by an analysis of first derivatives, identifying the precise point at which they begin to deviate significantly from zero (signifying no apparent slope in the performance curve). In the case of the neonatal module, the suggested threshold was around 30 items. We took a conservative approach, however, and increased this to 50 items. A limited range of symptoms applies to conditions that cause death in neonates and we wished to avoid eliminating items that might add important contextual information for automated diagnosis.

CSMF accuracy declines less rapidly than does CCC with progressive reduction in the number of items. This can be attributed to the finding that random allocation of deaths to different causes would result in CSMF accuracy of at least 63 % [25]. We have, however, chosen to show absolute values for CSMF accuracy, as it is this measure that will influence the use of CSMFs derived from VAs as the basis for public health policy development.

We would argue that the different forms of the VAI and the different methods of data collection and analysis need to be tailored for use in particular circumstances. Here, we have described an approach which should be invaluable for the collection of vital statistics.

## Conclusions

In this study, we developed a shortened VAI using a systematic approach and assessed its performance when administered by means of hand-held electronic tablets and analyzed using the Tariff 2.0 automated method. We demonstrated that, where the true cause of death is known, it is possible to reduce the length of a VA questionnaire by 40 % or more without a significant drop in performance. We have also shown that many of the advantages of the open-ended narrative in improving performance can be retained by use of an on-screen checklist. The reduced VAI developed has great utility to estimate COD data in civil registration and to calculate CSMFs in populations reducing the burden of time and resources required for data collection and analysis.

VA questionnaires have been constructed to elicit as much useful diagnostic information as possible for as many causes as possible for which symptomatic information is likely to be meaningful. Very little attention has been paid to the length of the interview or to the possible effects of interviewer/interviewee fatigue. If VA methods are to be useful beyond research settings to provide the essential intelligence on population cause of death patterns that governments and donors need, then it is critically important that they be rapidly integrated into national civil registration and vital statistics systems and routinely applied to all out-of-hospital deaths that are reported. New automated diagnostic methods and data collection platforms using tablets or mobile phones are now available for widespread use in civil registration systems. That utility will be even more enhanced if shorter VA questionnaires, such as the one reported here, are applied to cut interview time in half without any loss of diagnostic accuracy.

## Additional files

Additional file 1:
**Gold standard clinical diagnosis criteria.** (DOCX 117 kb) Additional file 2:
**Dataset composition.** (DOCX 120 kb) Additional file 3:
**Shortened PHMRC VAI.** (DOCX 133 kb) Additional file 4:
**Sensitivity by cause for each module in full and shortened PHMRC VAI.** (XLSX 15 kb) Additional file 5:
**Specificity by cause for each module in full and shortened PHMRC VAI.** (XLSX 14 kb) Additional file 6:
**Chance-corrected concordance by cause for full Population Health Metrics Research Consortium verbal autopsy instrument (PHMRC VAI) as compared to the shortened PHMRC VAI by type of text items included in the analysis.** (XLSX 15 kb)

## Abbreviations

CCC: Chance-corrected concordance
COD: Cause of death
CSMF: Cause-specific mortality fraction
PCVA: Physician certified verbal autopsy
PHMRC: Population Health Metrics Research Consortium
VA: Verbal autopsy
VAI: Verbal autopsy instrument
