# Supplementary material for: A shortened verbal autopsy instrument for use in routine mortality surveillance systems
Source: BMC Med. 2015 Dec 16;13:302. doi: 10.1186/s12916-015-0528-8 (PMC4681088; doi:10.1186/s12916-015-0528-8)
Supplement: Additional file 1: — Gold standard clinical diagnosis criteria. (DOCX 117 kb) [file 12916_2015_528_MOESM1_ESM.docx]

# Additional file 1: Gold standard clinical diagnosis criteria

**GC13 Adult Gold Standard Diagnoses**

Level 1 = Diagnosis of a particular condition with the highest level of certainty possible for that condition, consisting of either an appropriate laboratory test or x-ray with positive findings and/or medically observed and documented appropriate illness sign(s).

Level 2A = Diagnosis of a particular condition with a high level of certainty, consisting of medically observed and documented appropriate illness or sign(s).

Level 2B= Diagnosis of a particular condition with reasonable certainty but not meeting Level 1 or Level 2A criteria; this category was developed especially for cancer and HIV diagnoses where records are not available.

Level 3 = Cases which would be considered for a gold standard diagnosis but do not meet gold standard criteria: “possible gold standard cases.” This level is designed to exclude possible gold standard cases from the residual categories; no VAs should be collected for any level 3B causes of death.

**Notes:**

1. To be acceptable, illness signs must be observed and documented in a medical record by a physician or clinical officer, unless otherwise specifically noted.

2. Level 1 diagnosis should be the standard used for all gold standard cases. Only if it proves impossible to gather enough cases of a particular condition is it allowable to use the Level 2 diagnosis. For all causes, an autopsy report is acceptable as a gold standard confirmation. (Details are provided below.)

3. Residual Categories: In addition to the priority gold standard causes listed below, the data analysis method requires us to collect a sample of deaths from non-priority causes. These deaths will be grouped into residual categories. Thus, the residual categories will include deaths that occur from non-priority causes, clustered according to Global Burden of Disease cause spans to allow for a balanced distribution of residual causes in the data. The purpose of the Level 3 diagnosis is to prevent possible gold standard deaths from inclusion in the residual categories. If there is a suspicion that the death may be due to one of the priority gold standards, the death does not qualify for the residual category and should be excluded.

4. Several diagnoses are commonly associated with gold standard causes of deaths (co-morbid conditions) or represent a terminal process important for diagnosis. Criteria for co-morbid conditions are included in Appendix A. Criteria for terminal conditions are included in Appendix B.

**I. Adult Infectious Diseases**

**AIDS (A)**

Level 1 Positive for HIV on ELISA test, or Western Blot, or positive for two HIV rapid tests (based on the local standard of care)

PLUS one of the following:

- Extrapulmonary cryptococcosis including meningitis
- Disseminated non-tuberculosis mycobacteria infection
- Progressive multifocal leucoencephalopathy
- Candida of trachea, bronchi or lungs
- Cryptosporidiosis
- Isosporiasis
- Visceral herpes simplex infection
- Cytomegalovirus infection (retinitis of an organ other than liver, spleen or lymph node)
- Any disseminated mycosis (e.g. histoplasmosis, coccidiomycosis)
- Recurrent non-typhoidal salmonella septicaemia
- Lymphoma (cerebral or B-cell non-Hodgkin)
- Invasive cervical carcinoma
- HIV wasting syndrome
- Pneumocystis pneumonia
- Chronic herpes simplex infection (orolabial, genital or anorectal of more than one month’s duration)
- Esophageal candidiasis
- Kaposi’s sarcoma
- CNS toxoplasmosis

Level 2B Patient receiving treatment with ARV where the basis for the initial diagnosis is no longer available

Level 3 Clinical evidence of AIDS in the absence of HIV testing

AIDS smear positive for AFB

**AIDS with TB (A)**

Level 1 Both of the following:

- Positive for HIV on ELISA test, or Western Blot, or positive for two HIV rapid tests (based on the local standard of care)
- Culture positive for M.tuberculosis

Level 3 Evidence suggesting both TB and AIDS, including smear positive, but does not meet Level 1 criteria above

**Diarrhea (A)**

Level 1 Both of the following:

- Reported liquid or watery or loose stools 3+ times a day for at least 1 day
- Observed dehydration
- Observed liquid or semi-liquid or watery stools

Level 3 Death associated with diarrhea not meeting the above criteria (e.g. dehydration not observed)

**Dysentery (B)**

Level 1 Bloody diarrhea with one of the following:

- Isolation of *Shigella* from stools
- Identification of *E.histolytica* trophozoites in stools

Level 2A Bloody diarrhea with all of the following:

- Fever
- Gripping abdominal pain
- Tenesmus and/or rectal prolapse

Level 3 Death associated with bloody diarrhea not meeting the above criteria

**Malaria (B)**

Level 1 Thick malaria smear ≥5,000 parasites/microL or ≥150 parasites/200 wbcs

PLUS one of the following:

- Axillary or oral temperature ≥38.5⁰C
- Rectal temperature ≥39⁰C

Level 2 Both of the following:

- Rapid diagnostic test positive for malaria
- Strong clinical and epidemiological support for the diagnosis of malaria

Level 3B One of the following:

- Thick malaria smear ≥5,000 parasites/microL or ≥150 parasites/200 wbcs
- Fever not meeting the above criteria
- Lack of clinical and epidemiological support for determination of malaria (i.e. the presence of parasitemia alone is not sufficient for a diagnosis of death due to malaria)

**Pelvic Inflammatory Disease (B)**

Level 1 Each of the following:

- Lower abominal tenderness
- Unilateral or bilateral adnexal tenderness
- Cervical motion tenderness

PLUS one or more of the following:

- Abnormal cervical or vaginal discharge
- Axillary or oral temperature ≥38.5^o^C
- Rectal temperature ≥39^o^C
- Elevated ESR
- Elevated C-reactive protein
- Laboratory documentation of cervical infection due to N.gonorrhea or C.trachomatis

Level 3 Clinical diagnosis of PID failing to meet the above criteria

Excludes: Death within six weeks of puerperal sepsis or septic abortion (assign to maternal deaths)

Excludes: Deaths certified as being from ectopic pregnancy or appendicitis or other gastrointestinal causes of peritonitis (assign to these causes)

**Pneumonia (A)**

Level 1 Chest x-ray consistent with pneumonia (primary end-point consolidation or pleural effusion or other consolidation/infiltration)

PLUS two or more of the following:

- Respiratory rate >20 breaths/minute
- Abnormal breath sounds/Auscultations (i.e., signs of consolidation, crepitations)
- Axillary or oral temperature ≥38.5^0^  (rectal temperature ≥39^o^C)

Level 2A Two or more of the following:

- Respiratory rate >20/minute
- Abnormal breath sounds/Auscultations (i.e., signs of consolidation, crepitations)
- Axillary or oral temperature ≥38.5^0^ (rectal temperature ≥39^o^C)

Level 3 Death associated with cough, dyspnoea, and an acute febrile illness but not meeting the above

criteria

**Pulmonary tuberculosis (A)**

Level 1 Both of the following:

- Clinical history consistent with active pulmonary tuberculosis (e.g. cough, hemoptysis, weight loss, breathlessness, fever) during terminal illness
- Negative for HIV on ELISA test or two negative HIV rapid tests

PLUS one of the following:

- Two sputum smears positive for acid fast bacilli or culture positive for M.tuberculosis
- AFB smear positive or culture positive from material from other site (e.g. lymph nodes)

Level 2A Clinical history consistent with active pulmonary tuberculosis (e.g. cough, hemoptysis, weight loss, breathlessness, fever) during terminal illness

PLUS one of the following:

- Two sputum smears positive for acid fast bacilli or culture positive for M.tuberculosis
- AFB smear positive or culture positive from material from other site (e.g. lymph nodes)

Note: Level 2 diagnosis is acceptable only in areas with low HIV prevalence where HIV testing is not consistent with standard of care.

Level 3 Clinical history consistent with active pulmonary tuberculosis in the absence of laboratory

confirmation

Excludes chronic lung disease as a result of tuberculosis

**Other Infectious Diseases (B) (Residual Category)**

None of the above diagnoses.

Level 1 Laboratory confirmation of the infecting agent

Level 2 Clinical but not laboratory evidence

**II. Cancers**

**Breast Cancer (A)**

Level 1 One of the following:

- Operative specimen with histological confirmation
- Biopsy/fine needle aspiration cytology

Level 2A Both of the following:

- Mammography diagnosis
- Imaging evidence of metastases in bone, lung, etc. based on CT scan/MRI/x-rays

Level 2B Patient under treatment from a recognised cancer hospital or cancer unit for breast cancer in cases where the basis for the initial diagnosis is no longer available

Level 3 Clinical diagnosis of breast cancer in absence of above confirmation

**Cervical Cancer (A)**

Level 1 One of the following

- Biopsy
- Pap smear and clinical diagnosis of cervical cancer

Level 2A Visualization of cervical tumor by any means

Level 2B Patient under treatment from a recognised cancer hospital or cancer unit for cervical cancer in cases where the basis for the initial diagnosis is no longer available

**Colorectal Cancer (A)**

Level 1 One of the following:

- Operative specimen with histological confirmation
- Colonoscopy report with biopsy

Level 2B Patient under treatment from a recognised cancer hospital or cancer unit for colorectal cancer in cases where the basis for the initial diagnosis is no longer available OR

One of the following in isolation:

- - - - Colonoscopy report (gross)
      - CT/MRI evidence
      - Barium contrast radiology report
      - Surgeon’s report of laparotomy

Level 3 Clinical or imaging diagnosis in the absence of any visualisation of a tumor

**Esophageal Cancer (B)**

Level 1 One of the following:

- Operative specimen with histological confirmation
- Endoscopy report with biopsy

Level 2A One of the following:

- Endoscopy report (gross)
- CT/MRI evidence
- Barium contrast radiology report

Level 2B Patient under treatment from a recognised cancer hospital or cancer unit for esophageal cancer in cases where the basis for the initial diagnosis is no longer available

Level 3 Clinical: sensation of food or liquid sticking in the gullet in absence of further confirmation

**Leukemia (B)**

Level 1 Positive test on bone marrow biopsy

Level 2B Patient under treatment from a recognised cancer hospital or cancer unit for leukemia in cases where the basis for the initial diagnosis is no longer available OR

- Blood smear diagnosis in absence of marrow biopsy

Level 3 Clinical diagnosis in absence of hematological confirmation

**Primary Liver Cancer (A)**

Level 1 One of the following:

- Operative specimen with histological confirmation
- Biopsy specimen/fine needle aspiration cytology

Level 2B Patient under treatment from a recognised cancer hospital or cancer unit for primary hepatic cancer in cases where the basis for the initial diagnosis is no longer available OR

Level 3 One or more hepatic masses (clinical or ultrasound)

**Lung Cancer (A)**

Level 1 One of the following:

- Operative specimen with histological confirmation
- Bronchoscopy report with biopsy
- Sputum cytology/fine needle aspiration cytology

Level 2A One of the following:

- Imaging report (CT scan, MRI)
- Bronchoscopy diagnosis
- Chest radiograph with a single large mass evident

Level 2B Patient under treatment from a recognised cancer hospital or cancer unit for lung cancer in cases where the basis for the initial diagnosis is no longer available

Level 3 Chest radiograph with more than one mass OR Clinical diagnosis

**Lymphomas (B)**

Level 1 Positive test on lymph node biopsy

Level 2B Patient under treatment from a recognised cancer hospital or cancer unit for lymphoma in cases where the basis for the initial diagnosis is no longer available

Level 3 Clinical diagnosis in absence of biopsy

**Mouth/Oropharynx Cancer (B)**

Level 1 Operative/biopsy specimen with histological confirmation

Level 2B Patient under treatment from a recognised cancer hospital or cancer unit for oropharyngeal cancer in cases where the basis for the initial diagnosis is no longer available OR visualisation of the cancer by a cancer specialist in the absence of a biopsy

Level 3 Clinical report not meeting the above criteria

**Ovarian Cancer (B)**

Level 1 Operative specimen with histological confirmation

Level 2B Patient under treatment from a recognised cancer hospital or cancer unit for ovarian cancer in cases where the basis for the initial diagnosis is no longer available

Level 3 Clinical or imaging diagnosis in absence of histology

**Prostate Cancer (B)**

Level 1 Operative/biopsy specimen with histological confirmation

Level 2A Elevated PSA

PLUS one of the following:

- - - - Transrectal ultrasound morphology
      - Evidence of metastases

Level 2B Patient under treatment from a recognised cancer hospital or cancer unit for prostate cancer in cases where the basis for the initial diagnosis is no longer available OR Clinical diagnosis (nodular prostate) with metastases but no PSA test result

Level 3 Clinical diagnosis (nodular prostate) without metastases without PSA test result

**Stomach Cancer (A)**

Level 1 One of the following:

- Operative specimen with histological confirmation
- Endoscopy report with biopsy

Level 2A One of the following:

- Endoscopy report (gross)
- CT/MRI evidence
- Barium contrast radiology report

Level 2B Patient under treatment from a recognised cancer hospital or cancer unit for stomach cancer in cases where the basis for the initial diagnosis is no longer available

Level 3 Clinical or imaging diagnosis in absence of histology

**Uterine Cancer (B)**

Level 1 One of the following:

- Operative specimen with histological confirmation
- Biopsy
- Pap smear and clinical diagnosis of uterine cancer

Level 2A One of the following:

- Colposcopy/hysteroscopy report (gross)
- CT/MRI evidence of primary mass or lesion in the uterus

Level 2B Patient under treatment from a recognised cancer hospital or cancer unit for uterine cancer in cases where the basis for the initial diagnosis is no longer available

Level 3 Clinical diagnosis

**Other Defined Cancers (B) (Residual Category)**

Cancer from sites other than the above. Exclude any cancer deaths with clinical diagnosis only.

Level 1 Operative specimen with histological confirmation

Level 2B Patient under treatment from a recognised cancer hospital or cancer unit for cancer from a specific site other than the above in cases where the basis for the initial diagnosis is no longer available

**III. Adult Non-communicable**

**Asthma (B)**

Level 1 Both of the following:

- Evidence from spirometry or serial peak flow measurement of reversible airway obstruction > 20% AND
- Status asthmaticus: severe case of prolonged wheezing leading to death as assessed by a physician

Level 2A Both of the following:

- Status asthmaticus: recent onset of an episode of severe dyspnoea associated with wheeze leading to death
- Past history of episodic breathlessness and wheeze diagnosed as asthma

Level 3 Severe dyspnoea and wheeze leading to death in absence of clinical or laboratory evidence of

Asthma

**Cardiomyopathy (B) (as categorized under “Inflammatory Heart Disease”)**

Level 1 Echocardiograph showing a globally hypokinetic, dilated heart in the presence of CCF and in the absence of ischemia, valvular defects, or pericardial disease

Level 3 Radiographic and clinical evidence of a large, dilated heart with CCF in the absence of an

echocardiograph

**Cirrhosis (A)**

Level 1 Liver biopsy

Level 2A One of the following:

- Chronic liver failure supported by evidence of abnormal liver function tests and characteristic abnormalities on imaging
- Bleeding from confirmed oesophageal varices

Level 3 Clinical evidence of liver failure or upper GI bleeding without supporting laboratory confirmation or

imaging

**COPD (A)**

Level 1 Diagnosis established by one of:

- Spirometry diagnosis FEVI<70% with no response to bronchodilators
- Chest x-ray features hyperinflation, flat hemi-diaphragms, reduced peripheral vascular markings, presence of bullae in conjunction with clinical features of COPD

AND Terminal illness due to one of:

- Pneumonia
- Cor pulmonale
- Respiratory failure

Level 2B Diagnosis established by the following criteria:

- Productive cough and breathlessness for 3 or more months of the year for a minimum of 2 successive years

Level 3 Clinical diagnosis of COPD not meeting the above criteria

**Dementia (B)**

Level 1 One of the following:

- Clinical diagnosis of dementia by a neurologist, psychiatrist, or psychologist
- CT/MRI based evidence of multiple cerebral infarcts and clear history of progressive impairment of cognition in clear consciousness, manifested by memory loss, etc.

Level 2B Clinical diagnosis of dementia by a general practicioner

Level 3 Symptom history only; non-medical diagnosis

**Diabetes with Coma (B)**

Level 1 Both of the following:

- - - - Gold standard diagnosis of diabetes (see Appendix A)
      - Terminal features of diabetic ketoacidosis/hyperosmolar non ketotic coma

Level 3 Both of the following:

- Clinical history of diabetes not meeting the above laboratory criteria
- Terminal features of diabetic ketoacidosis/hyperosmolar non ketotic coma

**Diabetes with Renal Failure (B)**

Level 1 Gold standard diagnosis of diabetes (see Appendix A) plus gold standard diagnosis of renal failure

Level 3 Both of the following:

- Clinical history of diabetes not meeting the above laboratory criteria
- Biochemical evidence of renal failure

**Diabetes with Skin Infection/Sepsis (B)**

Level 1 Both of the following:

- Gold standard diagnosis of diabetes (see Appendix A)
- Diabetic foot ulcers, sacral ulcers, or other skin lesions characteristic of diabetes with septic shock (see Appendix B)

Level 3 Both of the following:

- Clinical history of diabetes not meeting the above laboratory criteria
- Presence of foot ulcers, sacral ulcers, or other skin lesions suggestive of diabetes

**Bacterial Endocarditis (as categorized under “Inflammatory Heart Disease”)**

Level 1 Both of the following:

- Echocardiagraph showing vegetations on heart valves
- Evidence of bacteremia from blood cultures

Level 2A At least three of the four following:

- Embolic phenomena (petechiae, retinal haemorrhages etc.)
- Evidence of bacteremia from blood cultures
- Cardiac murmur OR recent invasive procedures OR history of drug abuse parenterally
- Axillary or oral temperature ≥38.5⁰C (rectal temperature ≥39⁰C)

Level 3 Cardiac murmur with fever

Epilepsy (B)

Level 1 Both of the following

- Status epilepticus: repeated seizures leading to death either from airway obstruction or brain damage observed and documented by a clinician, in the absence of evidence of an underlying cause such as: intracranial space occupying lesion, cerebrovascular disease, connective tissue disorders, metabolic derangements, or CNS infections
- Past history of seizures

Level 3 Seizures before death not meeting the above criteria

IHD – Acute Myocardial Infarction (A)

Level 1 Evidence of acute MI within 3 months preceding death based upon one or more of the following:

- Cardiac perfusion scan
- ECG changes
- Documented history of CABG or PTCA or stenting
- Coronary angiography
- Enzyme changes (any troponin elevation or CK-MB isoenzyme elevation>2 times the upper limit of normal) in the context of myocardial ischemia

Level 2A Clinical evidence of the following:

- Sudden death within six hours of the onset of characteristic shock (see Appendix B) and chest pain when the case has been witnessed by a physician

Level 3 Sudden death associated with chest pain not meeting Level 2 criteria

IHD – Congestive Heart Failure (A)

Level 1 Both of the following:

- Documented history of ischaemic/hypertensive heart disease including ECG changes
- Radiological evidence of pulmonary congestion

Level 3 Clinical diagnosis of congestive heart failure

**Pericarditis (B) (as categorized under “Inflammatory Heart Disease”)**

Level 1 One of the following:

- ECG changes and evidence of pericardial effusion on echocardiography
- Aspiration of fluid from the pericardium

Level 3 Clinical diagnosis of pericarditis

**Renal Failure (A)**

Level 1 No diabetes or other primary clinically identified cause of death

PLUS one of the following:

- - - - Renal biopsy showing evidence of glomerular/interstitial/tubular disease
      - Elevated blood urea nitrogen and/or creatinine

Level 3 Meets the above criteria but diabetes not excluded

**Stroke (A)**

Level 1 Both of the following:

- CT scan/MRI
  - - - Sudden onset of paralysis, coma

Level 2A Within the 28 days prior to death, rapidly developing signs of a focal or global loss of cerebral function lasting more than 24 hours (or leading to death) with no apparent cause other than that of vascular origin

**Other Specified Cardiovascular Diseases (B) (Residual Category)**

None of the above diagnoses. Exclude poorly defined conditions, e.g. “cardiac failure.”

**Other Specified Digestive Diseases (B) (Residual Category)**

None of the above diagnoses. Excludes poorly defined conditions, e.g. melena.

**Other Non-communicable diseases (B) (Residual Category)**

None of the above diagnoses.

**IV. Maternal**

A maternal death is the death of a woman during pregnancy or within 6 weeks of either abortion or birth. It is a death from any cause related to or aggravated by the pregnancy or its management, but not from accidental or incidental causes.

For the causes listed below, the specific cause must be confirmed by a physician or registered midwife or by laboratory tests.

**Anemia (B)**

Level 1 Both of the following:

- - - - Hemoglobin <3 AND
      - Clinical diagnosis of congestive heart failure

**Hemorrhage (B)**

Level 1 Shock (see Appendix B) following excessive blood loss from one of the following:

- Antepartum hemorrhage due either to placenta praevia or placental separation (abruption)
- Primary postpartum haemorrhage (within 24 hours of vaginal delivery) associated with the measured loss of 500mls of blood or more
- Secondary postpartum haemorrhage (more than 24 hours after delivery)
- Spontaneous or medically induced abortion

Level 2A Level 2A Shock (see Appendix B) following excessive blood loss from one of the following:

- Antepartum hemorrhage due either to placenta previa or placental separation (abruption)
- Primary PPH (within 24 hours of vaginal delivery) associated with significant blood loss clinically assessed as requiring transfusion or having received transfusion
- Secondary PPH (more than 24 hours after delivery)
- Spontaneous or medically induced abortion

**Sepsis (B)**

Level 1 All of the following:

- Pyrexia ≥38^o^C
- Lower abdominal tenderness and pain
- Offensive discharge
- Clinical evidence of Level 1 shock (see Appendix B)
- Positive blood culture

Level 2A All of the following:

- Pyrexia ≥38^o^C
- Lower abdominal tenderness and pain
- Offensive discharge
- Clinical evidence of Level 2 shock (see Appendix B)

**Eclampsia (B)**

Level 1 All of the following:

- BP ≥140/90 mm Hg at 20 weeks gestation or later
- Albuminuria ≥ 3+
- Seizures leading to death
- No history of epilepsy or other reason for seizures (e.g., malaria or other acute encephalopathy)

Level 2A All of the following:

- BP >=140/90 at 20 weeks gestation or later
- Seizures leading to death
- No history of epilepsy or other reason for seizures (e.g., malaria or other acute encephalopathy)

Note: Excludes epilepsy and hypertension from other specific causes

**Obstructed Labor (B)**

Level 1 Diagnosis established by all of the following:

- Prolonged labor (> 12 hours)
- Failure of the presenting part to descend
- Failure of the cervix to dilate fully
- Excessive moulding if the head is the presenting part

PLUS terminal illness due to one of:

- - - - Surgical diagnosis of uterine rupture
      - Level 1 Sepsis

Level 2A All of the following:

- Prolonged labor (>12 hours)
- Failure of presenting part to descend
- Failure of the cervix to fully dilate
- Excessive moulding if the head is the presenting part

PLUS terminal illness due to one of:

- - - - Uterine rupture established by clinical examination
      - Level 2A Sepsis (see Appendix B)

**Other Defined Causes of Death as a Consequence of Pregnancy (B) (Residual Category)**

None of the above diagnoses. Excludes death as a consequence of pregnancy where the immediate cause is undefined.

**V. Injuries**

The following causes are ALWAYS considered accidental: Bite of Venomous Animal, Drowning, Falls, Fires, Poisonings, Road Traffic. They are NEVER overlapping with Homicide or Suicide. If Homicide is indicated, it is the ONLY cause. If Suicide is indicated, it is the ONLY cause.

**Bite of Venomous Animal (B)**

Level 1 Third party written accounts: police report, coroner’s/autopsy report, hospital record, newspaper

account

**Drowning (B)**

Level 1 Third party written accounts: police reports, coroner’s/autopsy report, hospital record, newspaper

account

**Falls (B)**

Level 1 Third party written accounts: police reports, coroner’s/autopsy, hospital record, newspaper account

**Fires (B)**

Level 1 Third party written accounts: police reports, coroner’s/autopsy, hospital record, newspaper account

**Poisonings (B)**

Level 1 Third party written accounts: police reports, coroner’s/autopsy, hospital record, newspaper account

**Road Traffic (B)**

Level 1 Third party written accounts: police reports, coroner’s/autopsy, hospital record, newspaper account

**Homicide (B)**

Level 1 Third party written accounts: police reports, coroner’s/autopsy, hospital record, newspaper account

**Suicide (B)**

Level 1 Third party written accounts: police reports, coroner’s/autopsy, hospital record, newspaper account

**Other Injuries (B) (Residual category)**

None of the above diagnoses.

Level 1 Third party written accounts: police reports, coroner’s/autopsy, hospital record, newspaper account

**Appendix A. Co-morbid Conditions**

If one of these conditions is present in addition to the primary gold standard cause of death, it should be noted in the data.

Gold standard conditions associated with the underlying cause of death and the following two conditions should be included as co-morbid conditions.

**Diabetes**

Screening for Diabetes in a healthy person:

Level 1 Screening one of:

- Fasting glucose ≥7.0 mmol/L (≥126 mg/dl)
- Oral glucose tolerance test, 2 hour glucose ≥11.1 mmol/L (≥200 mg/dl)
- HbA_1c_>6.5 mg/dl

Screening for diabetes in a sick person admitted to the hospital:

Level 1 Hospitalization for a complication of diabetes and no documented history of diabetes:

- Random glucose ≥11.1 mmol/L (≥200 mg/dl) on at least two occasions not influenced by recent meal or intravenous glucose

Hospitalization for a complication of diabetes a documented history of diabetes:

- Random glucose ≥11.1 mmol/L (≥200 mg/dl) on at least one occasion not influenced by recent meal or intravenous glucose

**Hypertension**

Level 1 One of the following:

- Systolic BP ≥ 140 mm Hg
- Diastolic BP ≥ 90 mm Hg

**Appendix B. Common Terminal Condition(s)**

This appendix is meant to detail conditions that are common across multiple priority gold standard causes of death, to help clarify diagnosis of such conditions. Shock is the only common terminal condition listed at this time.

**Shock**

Level 1 Three of the following:

- Obtunded
- Heart rate > 100
- Respiratory rate > 22
- Hypotension (systolic BP <100 mm Hg or a 30 mm fall in baseline BP
- Urine output <0.5 mL/Kg/hour

Level 2A Clinical diagnosis of shock

**GC13 Neonatal and Child Gold Standard Diagnoses**

Level 1 = Diagnosis of a particular condition with the highest level of certainty possible for that condition, consisting of either an appropriate laboratory test or x-ray with positive findings and/or medically observed and documented appropriate illness sign(s)

Level 2A = Diagnosis of a particular condition with a high level of certainty, consisting of medically observed and documented appropriate illness sign(s).

Level 2B= Diagnosis of a particular condition with reasonable certainty but not meeting Level 1 or Level 2A criteria: this category was developed especially for cancer and HIV diagnoses where records are not available

Level 3 = Cases which would be considered for a gold standard diagnosis but do not meet gold standard criteria: “possible gold standard cases.” This level is designed to exclude possible gold standard cases from the residual categories; no VAs should be collected for any level 3 causes of death.

**Notes:**

1. To be acceptable, illness signs must be observed and documented in a medical record by a physician, clinical officer or, in the case of stillbirths, preterm delivery or birth asphyxia, by a midwife.

2. Level 1 diagnosis should be the standard used for all gold standard cases. Only if it proves impossible to gather enough cases of a particular condition is it allowable to use the Level 2 diagnosis. For all causes, an autopsy report would be acceptable as a gold standard confirmation. (Details are provided below.)

3. Residual Categories (Appendix A): In addition to the priority gold standard causes listed below, the data analysis method requires us to collect a sample of deaths from non-priority causes. These deaths will be grouped into residual categories. Thus, the residual categories will include deaths that occur from non-priority causes, clustered according to Global Burden of Disease cause spans to allow for a balanced distribution of residual causes in the data. The purpose of the Level 3 diagnosis is to prevent possible gold standard deaths from inclusion in the residual categories. If there is a suspicion that the death may be due to one of the priority gold standards, the death does not qualify for the residual category and should be excluded.

4. Several pediatric conditions commonly occur together and may have additive or synergistic effects in causing death. These “co-morbid” conditions are listed at the end of the neonatal and childhood sections.

NEONATAL (Age < 28 days)

1. Neonatal Conditions

Birth Asphyxia (A)

Level 1 Each of the following:

- Failure both to breathe spontaneously and to cry at birth
- No major congenital abnormality
- Not a stillbirth (one or more signs of life at birth like pulse or movement)

PLUS one of the following in the 24 hours after birth:

- - - - Not feeding
      - Hypotonia
      - Seizures
      - Needed and failed resuscitation at birth

Congenital Malformation (A)

Level 1 Both of the following:

- Congenital malformation that is externally visible OR established by an imaging study
- Congenital malformation that is assessed by the study physician to have been the cause of death

*Examples:* Esophageal atresia; Gastroschisis; Hydrocephalus; Hypertrophic pyloric stenosis; Imperforate anus; Intestinal obstruction; Omphalocele

Level 3 Death assessed to have been caused by an internal congenital malformation that was not established by an imaging study

Meningitis (A) (as categorized under “Serious Infection”)

Level 1 Positive lumbar puncture, defined as one of the following:

- Any bacteria seen on gram stain of CSF
- >20 leukocytes/mm^3^ CSF with >80% PMNs
- Positive latex agglutination test of CSF
- Positive CSF culture

Level 3 Clinical diagnosis of meningitis with no lumbar puncture performed

Neonatal Tetanus (A)

Level 1 All of the following:

- Age at illness onset >2 days
- Not able to open mouth
- Rigidity

PLUS one of the following:

- Opisthotonus
- Spasms
- Convulsions

Pneumonia (A) (as categorized under “Serious Infection”)

Level 1 Chest x-ray showing primary end-point consolidation, pleural effusion or other consolidation/infiltration

PLUS two or more of the following:

- Respiratory rate >70/minute
- Severe lower chest indrawing
- Abnormal breath sounds (i.e., grunting, decreased breath sounds, crepitations)
- Rectal temperature >38^0^C or <36^0^C
- Oral or Axillary temperature >37.5^0^C or <35.5^0^C

Level 3 An acute febrile illness or hypothermia with cough and dyspnea, but not meeting the above criteria

Preterm Delivery (without Respiratory Distress Syndrome) (A)

Level 1 Not a stillbirth

PLUS one of the following:

- Birth at <33 weeks gestation (based on birth date minus the mother’s reported date of her last menstrual period)
- A physician or clinical officer’s Ballard gestational age assessment of <33 weeks (for hospital births)

PLUS one of the following:

- Chest x-ray negative for the characteristic “ground glass” appearance of RDS
- Death from another medically documented neonatal condition

Level 3 Meet all the above criteria, except gestational age = 33-36 weeks

**Preterm Delivery with Respiratory distress syndrome (RDS) (B)**

Level 1 Chest x-ray positive for characteristic “ground glass” appearance

PLUS one of the following:

- - - - - Preterm delivery: Birth at <33 weeks gestation, based on:

Birth date minus the mother’s reported date of her last menstrual period

A physician or clinical officer’s Ballard gestational age assessment <33 weeks

- - - - - Not preterm delivery: Birth at 33-36 weeks gestation, based on:

Birth date minus the mother’s reported date of her last menstrual period

A physician or clinical officer’s Ballard gestational age assessment 33-36 weeks

PLUS two or more of the following:

- - - - - Respiratory rate >70/minute
        - Central cyanosis (dusky, bluish lips or mucus membranes)
        - Severe lower chest wall indrawing
        - Grunting
        - Nasal flaring

Level 3 Death of a newborn <37 weeks gestation that breathed spontaneously at birth, from a non-hypothermic illness with tachypnea but not meeting the above criteria

Sepsis (A) (as categorized under “Serious Infection”)

Level 1

Positive laboratory criteria, including one of the following:

- Positive blood culture
- I:T (immature:total) neutrophil ratio >0.2

PLUS one of the following:

- Rectal temperature >38^o^C or <36^0^C
  - - - Oral or Axillary temperature >37.5^0^C or <35.5^0^C

PLUS one of the following:

- Irritability/agitated
- Abnormally sleepy, difficult to wake, lethargic or reduced to no spontaneous movement
- Unconscious
- Absent or weak cry
- Absent or weak suck
- Respiratory distress: respiratory rate >70/minute, severe lower chest indrawing, or grunting
- Mottled/cyanotic and cool extremities
- Reduced blood pressure

Level 3: Children who meet the Level 1 criteria but for whom either (1) both a blood culture and an I:T neutrophil ratio were obtained and both were negative, or (2) only an I:T neutrophil ratio was obtained and it was negative.

**Sepsis with local bacterial infection (B)**

Level 1 Level 1 Sepsis (A)

PLUS one of the following:

- Red umbilicus extending to the skin
- Purulent umbilicus
- Skin pustules
- Bullae
- Ulcer
- Furuncle (boil)
- Cellulitis

Level 3 Level 3 Sepsis (A) PLUS one of the above listed local bacterial infections

Stillbirth (A)

Level 1 Birth of a fetus without any sign of life, including movement of voluntary muscles, spontaneous breathing, cry or pulsation of the umbilical cord

PLUS one of the following:

- Birth at >28 weeks gestation (based on birth date minus the mother’s reported date of her last menstrual period)
- Birth weight >1,000 grams

Level 3 Birth of a fetus without any sign of life (as above)

PLUS one of the following:

- Birth at 22-27 weeks gestation (based on birth date minus the mother’s reported date of her last menstrual period)
- Birth weight 500-999 grams

II. Neonatal Co-morbid Conditions

Preterm delivery (without RDS) AND Birth asphyxia (B)

Level 1 Level 1 Preterm delivery (without RDS) PLUS Level 1 Birth asphyxia

Level 3 Level 3 Preterm delivery (without RDS) PLUS Level 3 Birth asphyxia

Preterm delivery (with or without RDS) AND Sepsis (B)

Level 1 Level 1 Preterm delivery (with or without RDS) PLUS Level 1 Sepsis

Level 3 Level 3 Preterm delivery (with or without RDS) PLUS Level 3 Sepsis

Preterm delivery (without RDS) AND Sepsis AND Birth asphyxia (B)

Level 1 Level 1 Preterm delivery (without RDS) PLUS Level 1 Sepsis AND Level 1 Birth asphyxia

Level 3 Level 3 Preterm delivery (without RDS) PLUS Level 3 Sepsis AND Level 3 Birth asphyxia

CHILDHOOD (Age ≥ 28 days and < 12 years)

I. Childhood Infectious Diseases

AIDS (A)

Level 1 No Level 1 TB

PLUS one of the following:

- Age at death <18 months and positive child HIV DNA PCR
- Age at death >18 months and positive child ELISA

PLUS one of the following:

- Age at death <12 months and CD4 count <20%
- Age at death 12-59 months and CD4 count <15%
- Age at death <18 months and total lymphocyte count <2500/mm^3^
- Age at death >18 months and total lymphocyte count <1500/mm^3^

       PLUS one of the following:

- Unexplained severe wasting or severe malnutrition not adequately responding to standard therapy
- Pneumocystis pneumonia
- Recurrent severe presumed bacterial infections (e.g. empyema, pyomyositis, bone or joint infection, meningitis, but excluding pneumonia)
- Chronic herpes simplex infection (orolabial or cutaneous of more than one month’s duration)
- Kaposi’s sarcorma
- Oesophageal candidiasis
- CNS toxoplasmosis (outside the neonatal period)
- HIV encephalopathy

Level 2B Patient receiving treatment with ARV where the basis for the initial diagnosis is no longer available

Level 3 Both of the following:

- No Level 1, 2 or 3 TB
- Clinical evidence of AIDS

PLUS one or both of the following:

- No HIV testing
- No CD4 testing AND no total lymphocyte testing

AIDS with Tuberculosis (A)

Level 1 Level 1 TB

PLUS one of the following:

- Age at death <18 months and positive child HIV DNA PCR
- Age at death >18 months and positive child ELISA

PLUS one of the following:

- Age at death <12 months and CD4 count <20%
- Age at death 12-59 months and CD4 count <15%
- Age at death <18 months and total lymphocyte count <2500/mm^3^
- Age at death >18 months and total lymphocyte count <1500/mm^3^

Level 3 Both of the following:

- Level 3 TB: Clinical history consistent with active pulmonary TB without laboratory or radiographic evidence
- HIV positive OR CD4 or total lymphocyte count as above

Diarrhea (A)

Level 1 All of the following:

- Reported liquid or watery or loose stools 3+ times a day for at least 1 day
- Dehydration
  - - - Observed liquid or semi liquid or watery stools

Level 3 Reported liquid, watery or loose stools

PLUS one or both of the following:

- No dehydration
- No observed liquid, semi liquid or watery stools

Dysentery (A)

Level 1 All of the following:

- Reported liquid or watery or loose stools 3+ times a day
- Observed gross blood in the stools
- Fever

PLUS one of the following:

- Documented pediatric septic shock
- Documented hemolytic uremic syndrome
- Documented renal failure

Level 3 A febrile illness with observed gross blood in the stools, but without documented septic shock, hemolytic uremic syndrome or renal failure

Encephalitis (B)

Level 1 Positive lumbar puncture, defined as all of the following:

- >10 leukocytes/ mm^3^ and >50% lymphocytes
- No bacteria seen on gram stain of CSF
- No bacteria seen on CSF culture (if performed)
- Negative latex agglutination test of CSF (if performed)

Level 3 Clinical diagnosis of encephalitis with no lumber puncture performed

Hemorrhagic Fever (B)

Level 1 Evidence of internal bleeding

PLUS one of the following:

- Rectal temperature >38^o^C or <36^0^C
- Oral or Axillary temperature >37.5^0^C or <35.5^0^C

Level 3 Both of the following:

- Evidence of internal bleeding
- Fever or hypothermia not meeting the above criteria

Malaria (A)

Level 1 Thick malaria smear >10,000 parasites/microL

PLUS one of the following:

- Rectal temperature >38^o^C
- Oral or Axillary temperature >37.5^o^C

Level 2A Rapid diagnostic test positive

PLUS one of the following:

- Rectal temperature >38^o^C
- Oral or Axillary temperature >37.5^o^C

Level 3 A febrile illness with a positive thick malaria smear

PLUS one of the following:

- Thick malaria smear <10,000 parasites/microl
- Fever not meeting the above criteria

Measles (B)

Level 1 Positive measles-specific IgM antibodies

Level 2 A One of the following:

- Koplik spots
- Blotchy or confluent maculopapular rash on the face alone or most prominent on the face

PLUS one of the following

- Rectal temperature >38^0^C
- Oral or Axillary temperature >37.5^0^C

PLUS at least one of the following:

- Cough
- Coryza
- Conjunctivitis

PLUS death from diarrhea or pneumonia complications within 30 days of the acute rash illness

Level 3 An acute illness with rash and fever, but not meeting the above criteria

Meningitis (B)

Level 1 Positive lumbar puncture, defined as one of the following:

- Bacteria on gram stain of CSF
- >100 leukocytes/ mm^3^ CSF with >80% PMNs
- Positive latex agglutination test of CSF
- Positive CSF culture

Level 3 Clinical diagnosis of meningitis with no lumbar puncture performed

**Pertussis (B)**

Level 1 Both of the following:

- Culture positive for *B. Pertussis*
- An acute cough illness of any duration

OR both of the following:

- PCR positive for pertussis
- Meets the CDC clinical case definition (see below)

OR both of the following:

- Epidemiological link to a case confirmed by culture or PCR
- Meets the CDC clinical case definition (see below)

Level 2A Case meets the CDC clinical case definition: Cough >2 weeks

PLUS one of the following:

- Paroxysms of coughing
- Inspiratory “whoop”
- Posttusive vomiting

Level 3 An illness with prolonged cough not meeting the above criteria

Pneumonia (A)

Level 1 Chest x-ray consistent with pneumonia (primary end-point consolidation or pleural effusion or other consolidation/infiltration*)

PLUS any 2 or more of the following:

- Respiratory rate >50/minute
- Lower chest indrawing
- Abnormal breath sounds/auscultations (i.e., signs of consolidation, crepitations)
- Rectal temperature >38^0^ or oral/axillary temperature >37.5^0^

Level 2A Any 2 or more of the following:

- Respiratory rate >50/minute
- Lower chest indrawing
- Abnormal breath sounds/auscultations (i.e., signs of consolidation, crepitations)
- Rectal temperature >38^0^ or oral/axillary temperature >37.5^0^

Note: A negative chest x-ray does not disqualify a level 2A pneumonia diagnosis.

These symptoms capture acute lower respiratory infections but most will be pneumonia

Level 3 An acute febrile illness with cough and dyspnoea, but not meeting the above criteria

*As indicated in the WHO Standardization of interpretation of chest radiographs for the diagnosis of pneumonia in children. WHO, Pneumonia Vaccine Trial Investigators’ Group, 2001, Annex 4.

Sepsis (B)

Level 1 Positive laboratory criteria, defined as one of the following:

- Positive blood culture
- I:T (immature:total) neutrophil ratio >0.2

PLUS one of the following:

- Rectal temperature >38^o^C or <36^o^C
- Oral or Axillary temperature >37.5^o^C or <35.5^o^C

PLUS one of the following:

- Irritability/agitated
- Abnormally sleepy, difficult to wake, lethargic or reduced to no spontaneous movement
- Unconscious
- Respiratory distress: respiratory rate >50/minute or lower chest indrawing or grunting
- Mottled/cyanotic and cool extremities
- Reduced blood pressure

Level 2A Meets clinical criteria, including one of the following:

- Rectal temperature >38^o^C or <36^o^C
- Oral or Axillary temperature >37.5^o^C or <35.5^o^C

PLUS one of the following:

- Irritability/agitated
- Abnormally sleepy, difficult to wake, lethargic or reduced to no spontaneous movement
- Unconscious
- Respiratory distress: respiratory rate >50/minute or lower chest indrawing or grunting
- Mottled/cyanotic and cool extremities
- Reduced blood pressure

Note: A negative blood culture alone (when an I:T neutrophil ratio was not obtained) does not disqualify a level 2A sepsis diagnosis.

Level 3 Children who meet the Level 1 or Level 2 criteria but for whom either (1) both a blood culture and an I:T neutrophil ratio were obtained and both were negative, or (2) only an I:T neutrophil ratio was obtained and was negative.

**Sepsis with local bacterial infection (B)**

Level 1 Level 1 Sepsis

PLUS one of the following:

- Skin pustules
- Bullae
- Ulcer
- Furuncle (boil)
- Cellulitis

Level 2A Level 2A Sepsis

PLUS one of the following:

- Skin pustules
- Bullae
- Ulcer
- Furuncle (boil)
- Cellulitis

Level 3 Level 3 Sepsis PLUS one of the above listed local bacterial infections

Tuberculosis (B) (only Pulmonary TB)

Level 1 One of the following:

- Culture of (sputum or gastric aspirate) specimen positive for MTB
- Two (sputum or gastric aspirate) smears positive for AFB

OR one (sputum or gastric aspirate) smear positive for AFB and chest x-ray suggestive of active TB

Level 2A    Chest x-ray suggestive of active TB

PLUS one of the following:

- Cough >2 weeks
- Known family member with TB

Level 3 Prolonged cough and a known family member with TB, but without the above laboratory or radiographic evidence

1. **Childhood Co-morbid Conditions**

**Pneumonia AND Diarrhea (B)**

Level 1 Level 1 Pneumonia PLUS Level 1 Diarrhea

Level 3 Level 3 Pneumonia PLUS Level 3 Diarrhea

**III. Childhood Injuries**

**Bite of Venomous Animal (B)**

Level 1 Third party written accounts: police report, coroner’s/autopsy, hospital record, newspaper account

**Drowning (B)**

Level 1 Third party written accounts: police reports, coroner’s/autopsy, hospital record, newspaper account

**Falls (B)**

Level 1 Third party written accounts: police reports, coroner’s/autopsy, hospital record, newspaper account

**Fires (B)**

Level 1 Third party written accounts: police reports, coroner’s/autopsy, hospital record, newspaper account

**Poisonings (B)**

Level 1 Third party written accounts: police reports, coroner’s/autopsy, hospital record, newspaper account

**Road Traffic (B)**

Level 1 Third party written accounts: police reports, coroner’s/autopsy, hospital record, newspaper account

**Violent Death (B)**

Level 1 Third party written accounts: police reports, coroner’s/autopsy, hospital record, newspaper account

**Appendix A. Childhood Residual Diseases**

**Other Childhood Infectious Diseases (B)**

None of the above diagnoses.

Level 1 Laboratory confirmation of the infecting agent

Level 2A Clinical but not laboratory evidence

**Malignant neoplasms (B)**

None of the above diagnoses. Exclude any malignant neoplasms with clinical diagnosis only.

Level 1 Histological or haematological confirmation

**Cardiovascular diseases (B)**

None of the above diagnoses. Exclude any cardiovascular diseases with clinical diagnosis only.

Level 1 Diagnosis based on appropriate cardiac function and/or imaging (e.g., echocardiograph) study

PLUS clinical findings

**Respiratory diseases (B)**

None of the above diagnoses. Exclude any respiratory diseases with clinical diagnosis only.

Level 1 Diagnosis based on appropriate lung function and/or imaging study PLUS clinical findings

**Digestive diseases (B)**

None of the above diagnoses. Exclude any digestive diseases with clinical diagnosis only.

Level 1 Diagnosis based on surgical, specimens, or biopsy findings

Level 2A Diagnosis based on visualization (endoscopic or operative findings)

**Other defined causes of child deaths (B)**

Excludes all gold standard conditions and conditions defined by other residual categories. Includes other non-communicable diseases and other injuries.
