# Supplementary material for: A shortened verbal autopsy instrument for use in routine mortality surveillance systems
Source: BMC Med. 2015 Dec 16;13:302. doi: 10.1186/s12916-015-0528-8 (PMC4681088; doi:10.1186/s12916-015-0528-8)
Supplement: Additional file 2: — Dataset composition. (DOCX 120 kb) [file 12916_2015_528_MOESM2_ESM.docx]

# Additional file 2: Dataset composition
