# Supplementary material for: A shortened verbal autopsy instrument for use in routine mortality surveillance systems
Source: BMC Med. 2015 Dec 16;13:302. doi: 10.1186/s12916-015-0528-8 (PMC4681088; doi:10.1186/s12916-015-0528-8)
Supplement: Additional file 3: — Shortened PHMRC VAI. (DOCX 133 kb) [file 12916_2015_528_MOESM3_ESM.docx]

# Additional file 3: Shortened PHMRC VAI

**POPULATION HEALTH METRICS RESEARCH CONSORTIUM**

**SHORTENED VERBAL AUTOPSY INSTRUMENT**

**ADULT AND ADOLESCENT VERBAL AUTOPSY MODULE**

**INTERVIEW BEGINS**

*Instructions to interviewer: Introduce yourself and explain the purpose of your visit. Ask to speak to the mother or to another adult who was the deceased’s main caretaker during the illness that led to death. If this is not possible, arrange a time to revisit the household when the caretaker will be home. (see example below).*

“My name is [your name]. I am an interviewer with the Population Health Metrics Research Consortium project. I have been informed that a death has occurred in your household. I am very sorry to hear that a member of your household has passed away. Please accept my sympathies. For the purpose of improving health care, we are collecting information on all recent deaths in this area. I would like to talk to the mother or main caretaker of [the deceased’s name] and ask some questions about the events and any symptoms that [the deceased’s name] had during her/his illness before death.”

**SECTION 5: INJURIES AND ACCIDENT**

| adult_5_1 | Did ________ suffer from an injury or accident that led to his/her death? | 1. Yes  2. No  8. Refused to answer  9. Don’t know | ****  ****  ****  **** |
| --- | --- | --- | --- |
|  | *If “No”, refused to answer or don’t know is checked, go to Section 2.* | |  |
| adult_5_2 | What kind of injury or accident did ____________suffer from?  *Ask respondent each in sequence and mark all to which the respondent indicated “Yes.”* | 1. Road traffic crash/injury  2. Fall  3. Drowning  4. Poisoning  5. Bite or sting by venomous animal  6. Burn/fire  7. Violence (suicide, homicide, abuse)  8. Refused to answer  9. Other injury, specify________ | ****  ****  ****  ****  ****  ****  ****  **** |
| adult_5_3 | Was the injury or accident self-inflicted? | 1. Yes  2. No  8. Refused to answer  9. Don’t know | ****  ****  ****  **** |
| adult_5_4 | Was the injury or accident intentionally inflicted by someone else? | 1. Yes  2. No  8. Refused to answer  9. Don’t know | ****  ****  ****  **** |
|  | ***Go to Section 6: HEALTH RECORDS*** | |  |

**SECTION 1: HISTORY OF CHRONIC CONDITIONS OF THE DECEASED**

| adult_1_1 | Was ______ ever told by a health professional that he or she ever suffered from one of the following? | | |
| --- | --- | --- | --- |
| adult_1_1a | Asthma | 1. Yes  2. No  8. Refused to answer  9. Don’t know | ****  ****  ****  **** |
| adult_1_1c | Cancer | 1. Yes  2. No  8. Refused to answer  9. Don’t know | ****  ****  ****  **** |
| adult_1_1d | Tuberculosis | 1. Yes  2. No  8. Refused to answer  9. Don’t know | ****  ****  ****  **** |
| adult_1_1g | Diabetes | 1. Yes  2. No  8. Refused to answer  9. Don’t know | ****  ****  ****  **** |
| adult_1_1h | Epilepsy | 1. Yes  2. No  8. Refused to answer  9. Don’t know | ****  ****  ****  **** |
| adult_1_1i | Heart Disease | 1. Yes  2. No  8. Refused to answer  9. Don’t know | ****  ****  ****  **** |
| adult_1_1l | Stroke | 1. Yes  2. No  8. Refused to answer  9. Don’t know | ****  ****  ****  **** |
| adult_1_1m | COPD (Chronic Obstructive Pulmonary Disease) | 1. Yes  2. No  8. Refused to answer  9. Don’t know | ****  ****  ****  **** |
| adult_1_1n | AIDS | 1. Yes  2. No  8. Refused to answer  9. Don’t know | ****  ****  ****  **** |

**SECTION 2: SYMPTOM CHECKLIST**

| adult_2_1 | For how long was ______ ill before s/he died? | | 1. __ __ years   *Enter 99 if unknown*   1. __ __ months   *Enter 99 if unknown*   1. __ __ days   *Enter 99 if unknown*   1. __ __ hours   *Enter 99 if unknown*  8. Refused to answer  9. Don’t know | ****  **** |
| --- | --- | --- | --- | --- |
| adult_2_2 | Did _____ have a fever? | | 1. Yes  2. No  8. Refused to answer  9. Don’t know | ****  ****  ****  **** |
| adult_2_7 | Did _____ have a rash? | | 1. Yes  2. No  8. Refused to answer  9. Don’t know | ****  ****  ****  **** |
|  | *If “No” or “Don’t know” or “Refused to answer” go to adult_2_10* | | | |
| adult_2_8 | How many days did ______ have the rash? | | 1. __ __ days  *Enter 99 if unknown*  8. Refused to answer  9. Don’t know |    |
| adult_2_9 | Where was the rash located? | | 1. Face  2. Trunk  3. Extremities  4. Everywhere  5. Other location specify (___________)  8. Refused to answer  9. Don’t know | ****  ****  ****  ****  ****  ****  **** |
| adult_2_10 | Did ____ have sores? | | 1. Yes  2. No  8. Refused to answer  9. Don’t know | ****  ****  ****  **** |
|  | *If “No” or “Don’t know” or “Refused to answer” go to adult_2_13* | | |  |
| adult_2_11 | Did the sores have clear fluid or pus? | | 1. Yes  2. No  8. Refused to answer  9. Don’t know | ****  ****  ****  **** |
|  | | | | |
| adult_2_13 | Did ______ have an ulcer (pit) on the foot? | | 1. Yes  2. No  8. Refused to answer  9. Don’t know | ****  ****  ****  **** |
|  | *If “No” or “Don’t know” or “Refused to answer” go to adult_2_21* | | | |
| adult_2_14 | Did the ulcer ooze pus? | | 1. Yes  2. No  8. Refused to answer  9. Don’t know | ****  ****  ****  **** |
|  | *If “No” or “Don’t know” or “Refused to answer” go to adult_2_21* | | |  |
| adult_2_15 | For how many days did the ulcer ooze pus? | | 1. __ __ days  *Enter 99 if unknown*  8. Refused to answer  9. Don’t know |    |
| adult_2_21 | Did _____ have yellow discoloration of the eyes? | | 1. Yes  2. No  8. Refused to answer  9. Don’t know | ****  ****  ****  **** |
|  | *If “No” or “Don’t know” or “Refused to answer” go to adult_2_25* | | | |
| adult_2_22 | For how long did ______ have the yellow discoloration? | | 1. __ __ months  *Enter 99 if unknown*  2. __ __ days  *Enter 99 if unknown*  8. Refused to answer  9. Don’t know |    |
| adult_2_25 | Did _____ have puffiness of the face? | | 1. Yes  2. No  8. Refused to answer  9. Don’t know | ****  ****  ****  **** |
|  | *If “No” or “Don’t know” or “Refused to answer” go to adult_2_27* | | |  |
| adult_2_26 | For how long did ______ have puffiness of the face? | | 1. __ __ months  *Enter 99 if unknown*  2. __ __ days  *Enter 99 if unknown*  8. Refused to answer  9. Don’t know |    |
| adult_2_27 | Did _______ have general puffiness all over his/her body? | | 1. Yes  2. No  8. Refused to answer  9. Don’t know | ****  ****  ****  **** |
|  | | | | |
| adult_2_29 | Did _____ have a lump in the neck? | | 1. Yes  2. No  8. Refused to answer  9. Don’t know | ****  ****  ****  **** |
| adult_2_30 | Did _____ have a lump in the armpit? | | 1. Yes  2. No  8. Refused to answer  9. Don’t know | ****  ****  ****  **** |
| adult_2_31 | Did _____ have a lump in the groin? | | 1. Yes  2. No  8. Refused to answer  9. Don’t know | ****  ****  ****  **** |
| adult_2_32 | Did _____ have a cough? | | 1. Yes  2. No  8. Refused to answer  9. Don’t know | ****  ****  ****  **** |
|  | *If “No” or “Don’t know” or “Refused to answer” go to adult_2_36* | | |  |
| adult_2_33 | For how long did ______ have a cough? | | 1. __ __ months  *Enter 99 if unknown*  2. __ __ days  *Enter 99 if unknown*  8. Refused to answer  9. Don’t know |    |
| adult_2_34 | Did the cough produce sputum? | | 1. Yes  2. No  8. Refused to answer  9. Don’t know | ****  ****  ****  **** |
| adult_2_35 | Did _____ cough blood? | | 1. Yes  2. No  8. Refused to answer  9. Don’t know | ****  ****  ****  **** |
| adult_2_36 | Did _____ have difficulty breathing? | | 1. Yes  2. No  8. Refused to answer  9. Don’t know | ****  ****  ****  **** |
|  | *If “No” or “Don’t know” or “Refused to answer” go to adult_2_43* | | |  |
| adult_2_38 | Was the difficulty continuous or on and off? | | 1. Continuous  2. On and off  8. Refused to answer  9. Don’t know | ****  ****  ****  **** |
|  | | | | |
| adult_2_43 | Did _____ experience pain in the chest in the month preceding death? | | 1. Yes  2. No  8. Refused to answer  9. Don’t know | ****  ****  ****  **** |
|  | *If “No” or “Don’t know” or “Refused to answer” go to adult_2_47* | | |  |
| adult_2_44 | How long did the pain last? | | 1. Less than 30 minutes  2. 30 minutes to 24 hours  3. More than 24 hours  8. Refused to answer  9. Don’t know | ****  ****  ****  ****  **** |
| adult_2_47 | Did _____ have more frequent loose or liquid stools than usual? | | 1. Yes  2. No  8. Refused to answer  9. Don’t know | ****  ****  ****  **** |
| adult_2_50 | Was there blood in the stool? | | 1. Yes  2. No  8. Refused to answer  9. Don’t know | ****  ****  ****  **** |
|  | *If “No” or “Don’t know” or “Refused to answer” go to adult_2_52* | | |  |
| adult_2_51 | Was there blood in the stool up until death? | | 1. Yes  2. No  8. Refused to answer  9. Don’t know | ****  ****  ****  **** |
| adult_2_52 | Did _________ stop urinating? | | 1. Yes  2. No  8. Refused to answer  9. Don’t know | ****  ****  ****  **** |
| adult_2_53 | Did _____ vomit in the week preceding the death? | | 1. Yes  2. No  8. Refused to answer  9. Don’t know | ****  ****  ****  **** |
|  | *If “No” or “Don’t know” or “Refused to answer” go to adult_2_57* | | |  |
| adult_2_55 | Was there blood in the vomit? | | 1. Yes  2. No  8. Refused to answer  9. Don’t know | ****  ****  ****  **** |
| adult_2_56 | Was the vomit black? | | 1. Yes  2. No  8. Refused to answer  9. Don’t know | ****  ****  ****  **** |
|  | | | | |
| adult_2_57 | Did _____ have difficulty swallowing? | | 1. Yes  2. No  8. Refused to answer  9. Don’t know | ****  ****  ****  **** |
|  | *If “No” or “Don’t know” or “Refused to answer” go to adult_2_60* | | |  |
| adult_2_58 | For how long before death did ______ have difficulty swallowing? | | 1. __ __ months  *Enter 99 if unknown*  2. __ __ days  *Enter 99 if unknown*  8. Refused to answer  9. Don’t know |    |
| adult_2_59 | Was the difficulty with swallowing with solids, liquids, or both? | | 1. Solids  2. Liquids  3. Both  8. Refused to answer  9. Don’t know | ****  ****  ****  **** |
| adult_2_60 | Did ______ have pain upon swallowing? | | 1. Yes  2. No  8. Refused to answer  9. Don’t know | ****  ****  ****  **** |
| adult_2_61 | Did _____ have belly pain? | | 1. Yes  2. No  8. Refused to answer  9. Don’t know | ****  ****  ****  **** |
|  | *If “No” or “Don’t know” or “Refused to answer” go to adult_2_64* | | |  |
| adult_2_62 | For how long before death did ______ have belly pain? | | 1. __ __ hours  *Enter 99 if unknown*  2. __ __ days  *Enter 99 if unknown*  3. __ __ months  *Enter 99 if unknown*  8. Refused to answer  9. Don’t know |    |
| adult_2_63 | Was the pain in the upper or lower belly? | | 1. Upper belly  2. Lower belly  8. Refused to answer  9. Don’t know | ****  ****  ****  **** |
| adult_2_64 | Did _____have a more than usual protruding belly? | | 1. Yes  2. No  8. Refused to answer  9. Don’t know | ****  ****  ****  **** |
|  | *If “No” or “Don’t know” or “Refused to answer” go to adult_2_67* | | |  |
| adult_2_66 | How rapidly did _______ develop the protruding belly? | 1. Rapidly  2. Slowly  8. Refused to answer  9. Don’t know | | ****  ****  ****  **** |
| adult_2_67 | Did _____ have any mass in the belly? | 1. Yes  2. No  8. Refused to answer  9. Don’t know | | ****  ****  ****  **** |
|  | *If “No” or “Refused to answer” or “Don’t know”, go to question adult_2_72* | | |  |
| adult_2_68 | For how long before death did ______ have a mass in the belly? | 1. __ __ months  *Enter 99 if unknown*  2. __ __ days  *Enter 99 if unknown*  8. Refused to answer  9. Don’t know | |    |
| adult_2_72 | Did _____ have a stiff neck? | 1. Yes  2. No  8. Refused to answer  9. Don’t know | | ****  ****  ****  **** |
|  | *If “No” or “Refused to answer” or “Don’t know” go to question adult_2_74* | | |  |
| adult_2_73 | For how long before death did ______ have stiff neck | 1. __ __ months  *Enter 99 if unknown*  2. __ __ days  *Enter 99 if unknown*  8. Refused to answer  9. Don’t know | |    |
| adult_2_74 | Did ______ experience a period of loss of consciousness? | 1. Yes  2. No  8. Refused to answer  9. Don’t know | | ****  ****  ****  **** |
|  | *If “No” or “Refused to answer” or “Don’t know” go to question adult_2_82* | | |  |
| adult_2_75 | Did the period of loss of consciousness start suddenly or slowly? | 1. Suddenly  2. Slowly  8. Refused to answer  9. Don’t know | |  |
| adult_2_77 | Did it continue until death? | 1. Yes  2. No  8. Refused to answer  9. Don’t know | | ****  ****  ****  **** |
|  | | | | |
| adult_2_82 | Did _____ have convulsions?  *(Demonstrate)* | 1. Yes  2. No  8. Refused to answer  9. Don’t know | | ****  ****  ****  **** |
|  | *If “No” or “Refused to answer” or “Don’t know” go to question adult_2_85* | | |  |
| adult_2_83 | For how long before death did the convulsions last? | 1. __ __ minutes  *Enter 99 if unknown*  2. __ __ hours  *Enter 99 if unknown*  8. Refused to answer  9. Don’t know | |    |
| adult_2_84 | Did the person become unconscious immediately after the convulsions? | 1. Yes  2. No  8. Refused to answer  9. Don’t know | | ****  ****  ****  **** |
| adult_2_85 | Was _______ in any way paralyzed? | 1. Yes  2. No  8. Refused to answer  9. Don’t know | | ****  ****  ****  **** |
|  | *If “No” or “Refused to answer” or “Don’t know” go to section 3.* | | |  |
| adult_2_87 | Which were the limbs or body parts paralyzed?  *Read through the list in sequence and MARK ALL THAT APPLY* | 1. Right side (arm and leg)  2. Left side (arm and leg)  3. Lower part of the body  4. Upper part of the body  5. One leg only  6. One arm only  7. Whole body  11. Other (specify ____________)  8. Refused to answer  9. Don’t know | |  |

|  | ***If the deceased was female, then continue to Section 3: Questions for Women.***  ***If the deceased was male, then go to Section 4: Tobacco Use*** |  |
| --- | --- | --- |

**SECTION 3: QUESTIONS FOR WOMEN**

| adult_3_1 | Did ________ have any swelling or lump in the breast? | 1. Yes  2. No  8. Refused to answer  9. Don’t know | ****  ****  ****  **** |
| --- | --- | --- | --- |
| adult_3_2 | Did ________ have any ulcers (pits) in the breast?  *Show photo* | 1. Yes  2. No  8. Refused to answer  9. Don’t know | ****  ****  ****  **** |
|  | *Refer to gen_5_4.*  *If the decedent is under 16 years old go to question adult_3_3a*  *If the decedent is 16-50 years old go to question adult_3_4*  *If the decedent is over 50 years old go to question adult_3_3* | | |
| adult_3_3a | Did ______ ever have a period or mensturate? | 1. Yes  2. No  8. Refused to answer  9. Don’t know | ****  ****  ****  **** |
|  | *If “No” skip to adult_3_5* | |  |
| adult_3_3 | Had ________’s periods stopped naturally because of menopause? | 1. Yes  2. No  8. Refused to answer  9. Don’t know | ****  ****  ****  **** |
|  | *If “No” skip to adult_3_5* | | |
| adult_3_4 | Did _______ have vaginal bleeding after cessation of menstruation? (post-menopausal) | 1. Yes  2. No  8. Refused to answer  9. Don’t know | ****  ****  ****  **** |
| adult_3_5 | Did ______ have vaginal bleeding other than her period? (intermenstrual) | 1. Yes  2. No  8. Refused to answer  9. Don’t know | ****  ****  ****  **** |
| adult_3_6 | Was there excessive vaginal bleeding in the week prior to death? | 1. Yes  2. No  8. Refused to answer  9. Don’t know | ****  ****  ****  **** |
|  | *If “No” is the answer to adult_3_3a go to Section 4: Tobacco Use*  *If “Yes” is the answer to adult_3_3 go to Section 4: Tobacco Use* | | |
| adult_3_7 | At the time of death was her period overdue? | 1. Yes  2. No  8. Refused to answer  9. Don’t know | ****  ****  ****  **** |
|  | *If “No” or “Refused to answer” or “Don’t know” go to question adult_3_10* | |  |
| adult_3_8 | For how many weeks was her period overdue? | 1. __ __ weeks  *Enter 99 if unknown*  8. Refused to answer  9. Don’t know |    |
| adult_3_9 | Did she have a sharp pain in the belly shortly before death? | 1. Yes  2. No  8. Refused to answer  9. Don’t know | ****  ****  ****  **** |
| adult_3_10 | Was she pregnant at the time of death? | 1. Yes  2. No  8. Refused to answer  9. Don’t know | ****  ****  ****  **** |
|  | *If “No” or “Refused to answer” or “Don’t know”, question adult_3_17* | |  |
| adult_3_11 | For how many months was she pregnant? | 1. __ __ months  *Enter 99 if unknown*  8. Refused to answer  9. Don’t know |    |
| adult_3_12 | Did ______ die during an abortion? | 1. Yes  2. No  8. Refused to answer  9. Don’t know | ****  ****  ****  **** |
|  | *If “Yes”, skip to adult_3_19* | |  |
| adult_3_13 | Did bleeding occur while she was pregnant? | 1. Yes  2. No  8. Refused to answer  9. Don’t know | ****  ****  ****  **** |
| adult_3_14 | Did she have excessive bleeding during labour or delivery? | 1. Yes  2. No  8. Refused to answer  9. Don’t know | ****  ****  ****  **** |
| adult_3_15 | Did she die during labor or delivery?  *(“Labor” is the period of time by which contractions are less than 10 minutes apart.)* | 1. Yes  2. No  8. Refused to answer  9. Don’t know | ****  ****  ****  **** |
| adult_3_16 | For how long was she in labor? | 1. __ __ hours  *Enter 99 if unknown*  8. Refused to answer  9. Don’t know |    |
|  | *If answer to adult_3_15 is “Yes”, skip to next section* | |  |
| adult_3_17 | Did she die within 6 weeks of having an abortion? | 1. Yes  2. No  8. Refused to answer  9. Don’t know | ****  ****  ****  **** |
|  | *If “Yes”, skip to adult_3_19* |  |  |
| adult_3_18 | Did she die within 6 weeks of childbirth? | 1. Yes  2. No  8. Refused to answer  9. Don’t know | ****  ****  ****  **** |
|  | *If “No” or “Refused to answer” or “Don’t know”, skip to next Section 4: Tobacco Use* | |  |
| adult_3_19 | Did she have excessive bleeding after delivery or abortion? | 1. Yes  2. No  8. Refused to answer  9. Don’t know | ****  ****  ****  **** |

**SECTION 4: TOBACCO USE**

| adult_4_1 | Did _________ use tobacco? | 1. Yes  2. No  8. Refused to answer  9. Don’t know | ****  ****  ****  **** |
| --- | --- | --- | --- |
|  | *If “No” or “Refused to answer” or “Don’t know” go to Section 5: Health Records* | |  |
| adult_4_2 | What kind of tobacco did _____ use? | \| 1. Cigarettes  2. Pipe  3. Chewing Tobacco  4. Local form of Tobacco  5. Other (specify ___________)  8.Refused to answer  9. Don’t know \| \| --- \| |  |
|  | *If “Yes” to cigarettes, continue to adult_4_4. If “No” to cigarettes, go to Section 5: Health Records* | |  |
| adult_4_4 | How much chewing tobacco did ________ use daily? | 1. __ __ number  *Enter 99 if unknown*  8. Refused to answer  9. Don’t know |    |

**SECTION 6: HEALTH RECORDS**

| adult_6_1 | Was care sought outside the home while the deceased had this illness? | 1. Yes  2. No  8. Refused to answer  9. Don’t know |        |
| --- | --- | --- | --- |
|  | *If “No” or “Don’t know” or “Refused to answer” go to adult_6_4* | | |
| adult_6_2 | Where or from whom did you seek care?  *(CHECK ALL THAT APPLY)* | 1. Traditional Healer 2. Homeopath 3. Religious leader 4. Government Hospital 5. Governmental health center or clinic 6. Private Hospital 7. Community-based practitioner associated with health system 8. Trained birth attendant 9. Private physician 10. Pharmacy, drug seller, store, market 11. Other provider 12. Relative, friend (outside household)   88. Refused to answer  99. Don’t know |                            |
| adult_6_3 | *Record the name and address of the hospital, health center or clinic where the care was sought.* : |  |  |
| adult_6_4 | Do you have any health records that belonged to the deceased? | 1. Yes  2. No  8. Refused to answer  9. Don’t know |        |
|  | *If “No” or “Don’t know” or “Refused to answer” go to adult_6_10* | |  |
| adult_6_5 | Can I see the health records? | 1. Yes  2. No  8. Refused to answer  9. Don’t know |        |
|  | *If “No” or “Don’t know” or “Refused to answer” go to adult_6_10. If “Yes”, and respondent allows you to see the records, transcribe all the entries* | |  |
| adult_6_6 | *Record the dates of the two most recent visits from the health record*  *If not listed, mark 9999* | 1. _ _/_ _/_ _ _ _   dd mm yyyy     1. _ _/_ _/_ _ _ _   dd mm yyyy |  |
| adult_6_7 | Record the date of the last note  *Enter 9999 if unknown* | _ _/_ _/_ _ _ _  dd mm yyyy |  |
| adult_6_8 | *Transcribe the note:* |  |  |
| adult_6_9 | Was a death certificate issued? | 1. Yes  2. No  8. Refused to answer  9. Don’t know |        |
|  | *If “No” or “Don’t know” or “Refused to answer” go to question child_5_17* | | |
| adult_6_10 | Can I see the death certificate? | 1. Yes  2. No  8. Refused to answer  9. Don’t know |        |
|  | *If “No” go to question child_5_17* | | |
| adult_6_11 | *Record the immediate cause of death from the certificate.* |  |  |
| adult_6_12 | *Record the first underlying cause of death from the certificate.* |  |  |
| adult_6_13 | *Record the second underlying cause of death from the certificate.* |  |  |
| adult_6_14 | *Record the third underlying cause of death from the certificate.* |  |  |
| adult_6_15 | *Record the contributing cause(s) of death from the certificate.* |  |  |
|  | **END OF HEALTH RECORDS SECTION**  **GO TO SECTION 7: OPEN ENDED RESPONSE AND INTERVIEWER COMMENTS/OBSERVATIONS** | | |

**Section 7: Open Ended Response and Interviewer Comments/Observations Section**

*Instructions to the interviewer: Say to the respondent: "Thank you for the patient responses to this exhaustive set of questions. Could you please summarize, or tell us in your own words, any additional information about the illness and/or death of your loved one?"*

*To the interviewer: Listen to what the respondent tells you in his/her own words. Do not prompt except for asking whether there was anything else after the respondent finishes. If the respondent mentions any of the following words, mark "mentioned". Tell the respondent to stop and start again if they mention a word of interest, so you have time to mark it down.*

**Adult Checklist**

|  | **Key words** | **Mentioned** |
| --- | --- | --- |
|  | Chronic Kidney Disease |  |
|  | Dialysis |  |
|  | Fever |  |
|  | Heart Attack (AMI) |  |
| adult_7_1 | Heart Problems |  |
|  | Jaundice |  |
|  | Liver Failure |  |
|  | Malaria |  |
|  | Pneumonia |  |
|  | Renal (Kidney) Failure |  |
|  | Suicide |  |

**END OF INTERVIEW.**

**THANK RESPONDENT FOR PARTICIPATION**

**POPULATION HEALTH METRICS RESEARCH CONSORTIUM**

**SHORTENED VERBAL AUTOPSY INSTRUMENT**

**GENERAL INFORMATION MODULE**

| ***If deceased was less or equal to 28 days old, begin the Neonatal and Child VA module at***  ***Section 1: Background Section.***  ***If deceased was older than than 28 days and younger than 12 years, begin the Neonatal and Child VA module at Section 4: Child Injuries and Accidents Section.*** |
| --- |

**Section 4:** **CHILD INJURIES AND ACCIDENTS**

| child_4_47 | Did ______ suffer an injury or accident that led to death? | 1. Yes  2. No  8. Refused to answer  9. Don’t know |        |
| --- | --- | --- | --- |
|  | *If “No” “Don’t know” or “Refused to answer”, go to Section 1* | |  |
| child_4_48 | What kind of injury or accident did ____ suffer from?  (Read through the list in sequence and MARK ALL THAT APPLY)  *If other injury, specify in child_4_48a* | 1. Road traffic crash/ injury  2. Fall  3. Drowning  4. Poisoning  5. Bite or sting by venomous animal  6. Burn/Fire  7. Violence (suicide, homicide, abuse)  11. Other injury, specify (__________)  8. Refused to answer  9. Don’t know |                    |
| child_4_49 | Was the injury or accident intentionally inflicted by someone else? | 1. Yes  2. No  8. Refused to answer  9. Don’t know |        |
|  | ***Go to Section 5: HEALTH RECORDS*** | |  |

**SECTION 1: BACKGROUND**

| child_1_1 | Was the deceased a singleton or multiple birth*?  **If two or more children are born at the same time, it is counted as a multiple birth, even if one or more of the babies are born dead.* | 1. Singleton 2. Multiple   8. Refused to answer  9. Don’t know |        |
| --- | --- | --- | --- |
|  | *If child_1_1 is “Singleton” skip to 2.3.* | |  |
| child_1_2 | Was this the first, second, or later in the birth order? | 1. First 2. Second 3. Third or more   8. Refused to answer  9. Don’t know |          |
|  | *If mother is respondent, mark child_1_3 as “yes”.*  *If mother is not respondent, go to child_1_3* | |  |
| child_1_3 | Is the mother still alive? | 1. Yes 2. No |    |
|  | *If “Yes”, go to child_1_6.* | |  |
| child_1_4 | Did the mother die during or after the delivery? | 1. During 2. After   8. Refused to answer  9. Don’t know |        |
|  | *If “During” delivery, go to child_1_6.* |  |  |
| child_1_5 | How long after the delivery did the mother die?  *Less than 24 hours = 0 days.*  *Use 1 month = 28 days to determine the number of months.* | 1. __ __ days  *Enter 99 if unknown*  2. __ __ months  *Enter 99 if unknown*  8. Refused to answer  9. Don’t know |        |
| child_1_6 | Where was the deceased born? | 1. Hospital 2. Other health facility 3. On route to hospital or other health facility 4. Home 5. Other (specify _________)   8. Refused to answer  9. Don’t know |              |
| child_1_7 | At the time of the delivery what was the size of the deceased:  *Read the question and slowly read the first 4 choices. Respondent should hear all four choices and then respond.*  *(Show photos)* | 1. Very small 2. Smaller than usual   3. About average  4. Larger than usual  8. Refused to answer  9. Don’t know |            |
| child_1_8 | What was the weight of the deceased at birth? | 1. __ __ grams  *Enter 9999 if unknown*  2. __ __ kilograms  *Enter 999 if unknown*  8. Refused to answer  9. Don’t know |    |
| child_1_11 | Was the child born alive or dead? | 1. Alive 2. Dead   8. Refused to answer  9. Don’t know |        |
| child_1_12 | Did the baby ever cry? | 1. Yes  2. No  8. Refused to answer  9. Don’t know |        |
|  |  |  |  |
| child_1_13 | Did the baby ever move? | 1. Yes  2. No  8. Refused to answer  9. Don’t know |        |
| child_1_14 | Did the baby ever breathe? | 1. Yes  2. No  8. Refused to answer  9. Don’t know |        |
| child_1_15 | *INTERVIEWER ONLY: Refer to questions child_1_12, child_1_13, and child_1_14. If all three responses are “No” then check “Yes” below. Otherwise, check “No.”*  *Yes No* |  |  |
|  | ***If you answered “Yes” to child_1_15 (stillbirth), then go to child_1_16***  ***If you answered “No” to child_1_15 (live birth), go to child_1_20*** | |  |
|  |  | |  |

| child_1_16 | Were there any bruises or signs of injury on the baby’s body at birth? | 1. Yes  2. No  8. Refused to answer  9. Don’t know |        |
| --- | --- | --- | --- |
| child_1_17 | Was the baby’s body (skin and tissue) pulpy? | 1. Yes  2. No  8. Refused to answer  9. Don’t know |        |
| child_1_18 | Was any part of the baby physically abnormal at time of delivery? (for example: body part too large or too small, additional growth on body) | 1. Yes  2. No  8. Refused to answer  9. Don’t know |        |
|  | *If “No” or “Don’t know” or “Refused to answer” go to Section 3.* | |  |
| child_1_19 | What were the abnormalities?  *MARK ALL THAT APPLY (Show photos)* | 1. Head size very small at time of birth  2. Head size very large at time of birth  3. Mass defect on the back of head or  4. Other (Specify________)  80. Refused to answer  90. Do not know |            |
|  | ***After completing child_1_19, continue to Section 2: MATERNAL HISTORY.*** | |  |

|  | | | |
| --- | --- | --- | --- |
| child_1_20 | How old was the baby/child when the fatal illness started?  *(Less than 24 hours = 00 days. Use 1 month = 28 days to determine the number of months.)* | 1. __ __ days  *Enter 99 if unknown*  2. __ __ months  *Enter 99 if unknown*  3. __ __ years  *Enter 99 if unknown*  8. Refused to answer  9. Don’t know |    |
| child_1_21 | How long did the illness last?  *(Less than 24 hours = 00 days. Use 1 month = 28 days to determine the number of months.)* | 1. __ __ days  *Enter 99 if unknown*  2. __ __ months  *Enter 99 if unknown*  8. Refused to answer  9. Don’t know |    |
| child_1_22 | Where did the deceased die? | 1. Hospital 2. Other health facility 3. On route to hospital or other health facility 4. Home 5. Other (specify _________)   8. Refused to answer  9. Don’t know |              |
| **STOP.**  **If the child is less than or equal to 28 days old, continue to SECTION 2: MATERNAL HISTORY.**  **If the child is 29 days—11 years old, go to SECTION 3: INFANT AND CHILD DEATHS.** | | | |

**SECTION 2: MATERNAL HISTORY**

| child_2_1 | Was the late part of the pregnancy (defined as the last 3 months), labor, or delivery complicated by any of the following problems?  *(Read each complication and mark all that apply.)*  *(Read “the mother” if the mother is not the respondent.)* | 1. You *(the mother*) had convulsions 2. You *(the mother)* had high blood 3. You *(the mother)* had severe anemia 4. You *(the mother)* had diabetes   5. Child delivered not head first  6. Cord delivered first  7. Cord around child’s neck  10. Excessive bleeding  11.Fever during labor  12.No complications  8. Refused to answer  9. Don’t know |                        |
| --- | --- | --- | --- |
| child_2_2 | How many months long was the pregnancy? | 1. __ __ months  *Enter 99 if unknown*  8. Refused to answer  9. Don’t know |    |
| child_2_4 | Was the baby moving in the last few days before the birth? | 1. Yes  2. No  8. Refused to answer  9. Don’t know |        |
| child_2_5 | When did you *(the mother)* last feel the baby move?  *(Read “the mother” if the mother is not the respondent.)* | 1. __ __ hours before delivery  *Enter 99 if unknown*  2. __ __ days before delivery  *Enter 99 if unknown*  8. Refused to answer  9. Don’t know |    |
| child_2_9 | Was the liquor foul smelling? | 1. Yes  2. No  8. Refused to answer  9. Don’t know |        |
|  | | | |
| child_2_11 | Did you *(the mother)* receive any vaccinations since reaching adulthood including during this pregnancy?  *(Read “the mother” if the mother is not the respondent.)* | 1. Yes  2. No  8. Refused to answer  9. Don’t know |        |
|  | *If “No” or “Don’t know” or “Refused to answer” go to question 3.8* | |  |
| child_2_12 | How many doses? | 1. One 2. Two 3. Three 4. Four 5. Five or more   8. Refused to answer  9. Don’t know |              |
| child_2_17 | Was the delivery…?  *(Read the choices and mark ONE.)* | 1. Vaginal with forceps 2. Vaginal w/out forceps 3. Vaginal Don’t know 4. C-Section   8. Refused to answer  9. Don’t know |            |
|  | **STOP.**  **Refer back to question child_1_15. If you answered “Yes,” go to *Section 5: HEALTH RECORDS***  **If you answered “No,” and child is less than or equal to 28 days old continue to Section 3: NEONATAL DEATHS.** | | |

**SECTION 3: NEONATAL DEATHS**

| child_3_2 | Was any part of the baby physically abnormal at time of delivery? (for example: body part too large or too small, additional growth on body) | 1. Yes  2. No  8. Refused to answer  9. Don’t know |        |
| --- | --- | --- | --- |
|  | *If “No” or “Don’t know” or “Refused to answer” go to 4.3* | | |
| child_3_3 | What were the abnormalities?  *MARK ALL THAT APPLY*  *(Show photos)* | 1. Head size very small at time of birth  2. Head size very large at time of birth  3. Mass defect on the back of head or  11. Other (Specify: ___________)  8. Refused to answer  9. Don’t know |            |
| child_3_4 | Did the baby breathe immediately after birth? | 1. Yes  2. No  8. Refused to answer  9. Don’t know |        |
|  | *If “No” go to* child_3_6 |  |  |
|  | | | |
| child_3_5 | Did the baby have difficulty breathing? | 1. Yes  2. No  8. Refused to answer  9. Don’t know |        |
| child_3_6 | Was anything done to try to help the baby breathe at birth? | 1. Yes  2. No  8. Refused to answer  9. Don’t know |        |
| child_3_7 | Did the baby cry immediately after birth? | 1. Yes  2. No  8. Refused to answer  9. Don’t know |        |
|  | *If “Yes” go to* child_3_9 | |  |
| child_3_8 | How long after birth did the baby first cry?  *(MARK ONE)* | 1. Within 5 minutes   2. Within 6-30 minutes  3. More than 30 minutes  4. Never  8. Refused to answer  9. Don’t know |            |
|  | *If “Never” go to child_3_12* | |  |
| child_3_9 | Did the baby stop being able to cry? | 1. Yes  2. No  8. Refused to answer  9. Don’t know |        |
|  | *If “No” or “Don’t know” or “Refused to answer” go to question 4.10* | | |
| child_3_10 | How long before the baby died did the baby stop crying? | 1. Less than one day 2. One day or more   8. Refused to answer  9. Don’t know |        |
| child_3_12 | Did the baby ever suckle in a normal way? | 1. Yes  2. No  8. Refused to answer  9. Don’t know |        |
| child_3_17 | During the illness that led to death, did the baby have difficult breathing? | 1. Yes  2. No  8. Refused to answer  9. Don’t know |        |
|  | *If “No” or “Don’t know” or “Refused to answer” go to 4.13* | | |
| child_3_19 | For how many days did the difficult breathing last?  *(Less than 1 day= “00”)* | 1. __ __ days  *Enter 99 if unknown*  8. Refused to answer  9. Don’t know |    |
| child_3_20 | During the illness that led to death, did the baby have fast breathing? | 1. Yes  2. No  8. Refused to answer  9. Don’t know |        |
|  | *If “No” or “Don’t know” or “Refused to answer” go to child_3_24* | | |
| child_3_22 | For how many days did the fast breathing last?  *(Less than 1 day= “00”)* | 1. __ __ days  *Enter 99 if unknown*  8. Refused to answer  9. Don’t know |    |
| child_3_24 | During the illness that led to death, did the baby have grunting?  (*Demonstrate*) | 1. Yes  2. No  8. Refused to answer  9. Don’t know |        |
| child_3_25 | During the illness that led to death did the baby have spasms or convulsions? | 1. Yes  2. No  8. Refused to answer  9. Don’t know |        |
| child_3_26 | During the illness that led to death, did the baby have fever? | 1. Yes  2. No  8. Refused to answer  9. Don’t know |        |
| child_3_29 | During the illness that led to death, did the baby become cold to touch? | 1. Yes  2. No  8. Refused to answer  9. Don’t know |        |
| child_3_32 | During the illness that led to death, did the baby become lethargic, after a period of normal activity? | 1. Yes  2. No  8. Refused to answer  9. Don’t know |        |
| child_3_39 | During the illness that led to death, did the baby have ulcer(s) (pits)? | 1. Yes  2. No  8. Refused to answer  9. Don’t know |        |
| child_3_44 | During the illness that led to death, did he/she have more frequent loose or liquid stools than usual? | 1. Yes  2. No  8. Refused to answer  9. Don’t know |        |
| child_3_47 | During the illness that led to death, did he/she have yellow skin? | 1. Yes  2. No  8. Refused to answer  9. Don’t know |        |
|  |  |  |  |
| child_3_49 | Did the infant appear to be healthy and then just die suddenly? | 1. Yes  2. No  8. Refused to answer  9. Don’t know |        |

| **END OF NEONATAL DEATHS SECTION**  **GO TO SECTION 5: HEALTH RECORDS SECTION** |
| --- |

**SECTION 4: INFANT AND CHILD DEATHS**

| child_4_1 | During the illness that led to death, did ____________ have a fever? | 1. Yes  2. No  8. Refused to answer  9. Don’t know |        |
| --- | --- | --- | --- |
|  | *If “No” or “Don’t know” or “Refused to answer” go to child_4_6* | | |
| child_4_4 | How severe was the fever? | 1. Mild  2. Moderate  3. Severe  8. Refused to answer  9. Don’t know |          |
| child_4_6 | During the illness that led to death, did _____________ have more frequent loose or liquid stools than usual? | 1. Yes  2. No  8. Refused to answer  9. Don’t know |        |
|  | *If “No” or “Don’t know” or “Refused to answer” go to child_4_12* | | |
| child_4_7 | How many stools did __________ have on the day that loose or liquid stools were most frequent? | 1. __ __ stools  *Enter 99 if unknown*  8. Refused to answer  9. Don’t know |    |
| child_4_8 | How many days before death did the frequent loose or liquid stools start? | 1. Less than 24 hours  2. __ __ days  *Enter 99 if unknown*  8. Refused to answer  9. Don’t know |        |
|  | *If Less than 24 hrs, go to child_4_12* | | |
| child_4_9 | Did the frequent loose or liquid stools continue until death? | 1. Yes  2. No  8. Refused to answer  9. Don’t know |        |
|  |  |  |  |
| child_4_11 | Was there visible blood in the loose or liquid stools? | 1. Yes  2. No  8. Refused to answer  9. Don’t know |        |
| child_4_12 | During the illness that led to death, did the child have a cough? | 1. Yes  2. No  8. Refused to answer  9. Don’t know |        |
|  | *If “No” or “Don’t know” or “Refused to answer” go to child_4_16* | | |
| child_4_13 | For how many days did the cough last? | 1. __ __ days  *Enter 99 if unknown*  8. Refused to answer  9. Don’t know |    |
| child_4_14 | Was the cough very severe? | 1. Yes  2. No  8. Refused to answer  9. Don’t know |        |
| child_4_16 | During the illness that led to death, did _____________ have difficult breathing? | 1. Yes  2. No  8. Refused to answer  9. Don’t know |        |
|  | *If “No” or “Don’t know” or “Refused to answer” go to child_4_18* | | |
| child_4_17 | For how many days did the difficult breathing last? | 1. __ __ days  *Enter 99 if unknown*  8. Refused to answer  9. Don’t know |    |
| child_4_18 | During the illness that led to death, did _____________ have fast breathing? | 1. Yes  2. No  8. Refused to answer  9. Don’t know |        |
|  | *If “No” or “Don’t know” or “Refused to answer” go to child_4_23* | | |
| child_4_19 | For how many days did the fast breathing last? | 1. __ __ days  *Enter 99 if unknown*  8. Refused to answer  9. Don’t know |    |
|  | ***Note to Interviewer: If BOTH child_4_16 and child_4_18 are “No” go to child_4_25*** | | |
| child_4_23 | During the illness that led to death, did his/her breathing sound like grunting?  (*Demonstrate the sound*) | 1. Yes  2. No  8. Refused to answer  9. Don’t know |        |
| child_4_25 | Did ____________ experience any generalized convulsions or fits during the illness that led to death? | 1. Yes  2. No  8. Refused to answer  9. Don’t know |        |
| child_4_26 | Was _____________ unconscious during the illness that led to death? | 1. Yes  2. No  8. Refused to answer  9. Don’t know |        |
|  | *If “No” or “Don’t know” or “Refused to answer” go to child_4_28* | | |
| child_4_27 | How long before death did unconsciousness start? | 1. Less than 6 hours  2. 6-23 hours  3. 24 hours or more  8. Refused to answer  9. Don’t know |          |
| child_4_28 | Did ____________ have a stiff neck during the illness that led to death?  (*Demonstrate*) | 1. Yes  2. No  8. Refused to answer  9. Don’t know |        |
| child_4_29 | Did ____________ have a bulging fontanelle during the illness that led to death?  *(Show photo)* | 1. Yes  2. No  8. Refused to answer  9. Don’t know |        |
| child_4_30 | During the month before he/she died, did _____________ have a skin rash? | 1. Yes  2. No  8. Refused to answer  9. Don’t know |        |
|  | *If “No” or “Don’t know” or “Refused to answer” go to child_4_36* | | |
| child_4_33 | How many days did the rash last? | 1. __ __ days  *Enter 99 if unknown*  8. Refused to answer  9. Don’t know |    |
| child_4_36 | During the illness that led to death, did __________ have swollen legs or feet | 1. Yes  2. No  8. Refused to answer  9. Don’t know |        |
|  | *If “No” or “Don’t know” or “Refused to answer” go to 5.25* | | |
| child_4_37 | How long did the swelling last? | 1. __ __ days  *Enter 99 if unknown*  2. __ __ weeks  *Enter 99 if unknown*  8. Refused to answer  9. Don’t know |    |
| child_4_38 | During the illness that led to death, did ____________’s skin flake off in patches? | 1. Yes  2. No  8. Refused to answer  9. Don’t know |        |
| child_4_39 | Did ____________’s hair change in color to a reddish or yellowish color? | 1. Yes  2. No  8. Refused to answer  9. Don’t know |        |
| child_4_40 | Did ____________ have a protruding belly? | 1. Yes  2. No  8. Refused to answer  9. Don’t know |        |
| child_4_41 | During the illness that led to death, did __________ suffer from “lack of blood” or “pallor”? | 1. Yes  2. No  8. Refused to answer  9. Don’t know |        |
| child_4_42 | During the illness that led to death, did ____________ have swelling in the armpits? | 1. Yes  2. No  8. Refused to answer  9. Don’t know |        |
| child_4_43 | During the illness that led to death, did ____________ have a whitish rash inside the mouth or on the tongue? | 1. Yes  2. No  8. Refused to answer  9. Don’t know |        |
| child_4_44 | During the illness that led to death, did ­________ bleed from anywhere? | 1. Yes  2. No  8. Refused to answer  9. Don’t know |        |
| child_4_46 | During the illness that led to death, did he/she have areas of the skin that turned black? | 1. Yes  2. No  8. Refused to answer  9. Don’t know |        |
| **END OF INFANT AND CHILD DEATHS SECTION**  **GO TO SECTION 5: HEALTH RECORDS SECTION** | | | |

**SECTION 5: HEALTH RECORDS**

| child_5_1 | Was care sought outside the home while the deceased had this illness? | 1. Yes  2. No  8. Refused to answer  9. Don’t know |        |
| --- | --- | --- | --- |
|  | *If “No” or “Don’t know” or “Refused to answer” go to child_5_4* | | |
| child_5_2 | Where or from whom did you seek care?  *(CHECK ALL THAT APPLY)* | 1. Traditional Healer 2. Homeopath 3. Religious leader 4. Government Hospital 5. Governmental health center or clinic 6. Private Hospital 7. Community-based practitioner associated with health system 8. Trained birth attendant 9. Private physician 10. Pharmacy, drug seller, store, market 11. Other provider 12. Relative, friend (outside household)   88. Refused to answer  99. Don’t know |                            |
| child_5_3 | *Record the name and address of the hospital, health center or clinic where the care was sought.* : |  |  |
| child_5_4 | Do you have any health records that belonged to the deceased? | 1. Yes  2. No  8. Refused to answer  9. Don’t know |        |
|  | *If “No” or “Don’t know” or “Refused to answer” go to child_5_10* | |  |
| child_5_5 | Can I see the health records? | 1. Yes  2. No  8. Refused to answer  9. Don’t know |        |
|  | *If “No” or “Don’t know” or “Refused to answer” go to child_5_10. If “Yes”, and respondent allows you to see the records, transcribe all the entries* | |  |
| child_5_6 | *Record the dates of the two most recent visits from the health record*  *If not listed, mark 9999* | 1. _ _/_ _/_ _ _ _   dd mm yyyy     1. _ _/_ _/_ _ _ _   dd mm yyyy |  |
| child_5_7 | *Record the two most recent weights on those dates from the health record*  *Enter 9999 if unknown* | 1. grams _ _ _ _ 2. grams _ _ _ _ |  |
|  | | | |
| child_5_8 | Record the date of the last note  *Enter 9999 if unknown* | _ _/_ _/_ _ _ _  dd mm yyyy |  |
| child_5_9 | *Transcribe the note:* |  |  |
| child_5_10 | Was a death certificate issued? | 1. Yes  2. No  8. Refused to answer  9. Don’t know |        |
|  | *If “No” or “Don’t know” or “Refused to answer” go to question child_5_17* | | |
| child_5_11 | Can I see the death certificate? | 1. Yes  2. No  8. Refused to answer  9. Don’t know |        |
|  | *If “No” go to question child_5_17* | | |
| child_5_12 | *Record the immediate cause of death from the certificate.* |  |  |
| child_5_13 | *Record the first underlying cause of death from the certificate.* |  |  |
| child_5_14 | *Record the second underlying cause of death from the certificate.* |  |  |
| child_5_15 | *Record the third underlying cause of death from the certificate.* |  |  |
| child_5_16 | *Record the contributing cause(s) of death from the certificate.* |  |  |
| child_5_17 | Has the deceased’s (biological) mother ever been tested for “HIV”? | 1. Yes  2. No  8. Refused to answer  9. Don’t know |        |
|  | *If “No” or “Don’t know” or “Refused to answer” go to question child_5_19* | | |
| child_5_18 | Was the “HIV” test ever positive? | 1. Yes  2. No  8. Refused to answer  9. Don’t know |        |
| child_5_19 | Has the deceased’s (biological) mother ever been told she had “AIDS” by a health worker? | 1. Yes  2. No  8. Refused to answer  9. Don’t know |        |
|  | **END OF HEALTH RECORDS SECTION**  **GO TO SECTION 6: OPEN ENDED RESPONSE AND INTERVIEWER COMMENTS/OBSERVATIONS**  **If the child is less than or equal to 28 days, continue on to neonate_6_1**  **If the child is older than 28 days, continue on to child_6_1** | | |

**Section 6: Open Ended Response and Interviewer Comments/Observations Section**

*Instructions to the interviewer: Say to the respondent: "Thank you for the patient responses to this exhaustive set of questions. Could you please summarize, or tell us in your own words, any additional information about the illness and/or death of your loved one?"*

*To the interviewer: Listen to what the respondent tells you in his/her own words. Do not prompt except for asking whether there was anything else after the respondent finishes. If the respondent mentions any of the following words, mark "mentioned". Tell the respondent to stop and start again if they mention a word of interest, so you have time to mark it down.*

**If the child is less than or equal to 28 days, use the key words listed out in neonate_6_1**

**If the child is older than 28 days, use the key words listed out in child_6_1**

**Neonatal Checklist**

|  | **Key words** | **Mentioned** |
| --- | --- | --- |
|  | Asphyxia (lack of oxygen) |  |
|  | Incubator |  |
| neonate_6_1 | Lung Problems |  |
|  | Pneumonia |  |
|  | Preterm Delivery |  |
|  | Respiratory Distress |  |

**Child Checklist**

|  | **Key words** | **Mentioned** |
| --- | --- | --- |
|  | Abdomen |  |
|  | Cancer |  |
|  | Chicken pox |  |
|  | Dehydration |  |
| child_6_1 | Dengue fever |  |
|  | Diarrhea |  |
|  | Fever |  |
|  | Heart Problems |  |
|  | Jaundice (yellow skin or eyes) |  |
|  | Pneumonia |  |
|  | Rash |  |

**END OF INTERVIEW.**

**THANK RESPONDENT FOR PARTICIPATION**
